# Effect of Microwave Treatment in a High Pressure Microwave Reactor on Graphene Oxide Reduction Process—TEM, XRD, Raman, IR and Surface Electron Spectroscopic Studies

**DOI:** 10.3390/ma14195728

**Published:** 2021-09-30

**Authors:** Beata Lesiak, Grzegorz Trykowski, József Tóth, Stanisław Biniak, László Kövér, Neha Rangam, Artur Małolepszy, Leszek Stobiński

**Affiliations:** 1 Institute of Physical Chemistry, Polish Academy of Sciences, Kasprzaka 44/52, 01-224 Warsaw, Poland; neha.rangam@ichf.edu.pl; 2 Faculty of Chemistry, Nicolaus Copernicus University in Torun, Gagarina 7, 87-100 Torun, Poland; sbiniak@umk.pl; 3 Institute for Nuclear Research, P.O. Box 51, H-4001 Debrecen, Hungary; toth.jozsef@atomki.mta.hu (J.T.); lkover@atomki.mta.hu (L.K.); 4 Faculty of Chemical and Process Engineering, Warsaw University of Technology, Warynskiego 1, 00-645 Warsaw, Poland; artur.malolepszy@pw.edu.pl (A.M.); lstob50@hotmail.com (L.S.); 5 Nanomaterials Leszek Stobinski, 03-337 Warsaw, Poland

**Keywords:** graphene oxide (GO), reduced graphene oxide (rGO), structural, chemical properties

## Abstract

Reduced graphene oxide (rGO) was prepared by chemical reduction of graphene oxide (GO) (with a modified Hummers method) in aqueous solutions of hydrazine (N_2_H_4_), formaldehyde (CH_2_O), formic acid (HCO_2_H) accompanied by a microwave treatment at 250 °C (MWT) by a high pressure microwave reactor (HPMWR) at 55 bar. The substrates and received products were investigated by TEM, XRD, Raman and IR spectroscopies, XPS, XAES and REELS. MWT assisted reduction using different agents resulted in rGOs of a large number of vacancy defects, smaller than at GO surface C sp^3^ defects, oxygen groups and interstitial water, interlayer distance and diameter of stacking nanostructures (flakes). The average number of flake layers obtained from XRD and REELS was consistent, being the smallest for CH_2_O and then increasing for HCO_2_H and N_2_H_4_. The number of layers in rGOs increases with decreasing content of vacancy, C sp^3^ defects, oxygen groups, water and flake diameter. MWT conditions facilitate formation of vacancies and additional hydroxyl, carbonyl and carboxyl groups at these vacancies, provide no remarkable modification of flake diameter, what results in more competitive penetration of reducing agent between the interstitial sites than via vacancies. MWT reduction of GO using a weak reducing agent (CH_2_O) provided rGO of 8 layers thickness.

## 1. Introduction

Nowadays, a large number of methods of graphene production using “top-to-bottom” methods (chemical synthesis, mechanical, chemical and electrochemical exfoliation) and “bottom-to-top” methods (chemical vapor deposition, epitaxial growth, rapid thermal annealing, biomass pyrolysis) are being applied [1,2,3]. However, a large-scale mass production and commercialization of graphene synthesis would require the effective, low-cost, energy and time, environment friendly chemical procedures. Among those, the recently applied microwave (MW) assisted synthesis [4,5] and/or synthesis from a biomass [3] are challenging. The MW methods have been used in the field of carbon nanomaterials preparation, such as in exfoliation and intercalation of graphite [6,7,8,9,10], graphene oxide reduction [4,5,11,12,13,14,15,16], preparation of metal decorated carbon nanomaterials and other composites [16,17] and unzipping of nanofibers [18].

The mass production used for separating graphene layers from graphite leads to defected graphite of uncontrolled structural parameters [19], where chemical and structural properties affect the dielectric properties [20]. Mass production of graphene proceeds from exfoliated graphite intercalation compounds (GICs). The GIC, as a complex material, in which elements or molecules are intercalated between graphite layers, is produced using electrochemical and or chemical methods. The exfoliation of GIC results from the separation of graphene layers due to rapid evaporation of intercalates by a heating process, also using a microwave heating. The application of GIC to produce graphene requires increasing the distance between the graphite layers by weakening van der Waals interactions between the layers. Since intercalates provide stronger interaction between the layers, other solutions to weaken these interactions, e.g., solvents, lowering the energy of exfoliation, are frequently proposed. The exfoliation proceeds during electrochemical and/or chemical procedures, and also in different solvents, being accompanied by a microwave irradiation. The microwave energy using a microwave absorbent is transformed efficiently into heat. This microwave heating is applied as a fast-heating process and leads to a larger surface area and volume than in the conventional heating technique. Microwave irradiation accompanied by exfoliation in a solvent also facilitates the penetration of solvent molecules, preventing re-aggregation of exfoliated graphene layers, which results in increasing graphene yield. The interaction of electromagnetic radiation such as microwaves or infra-red radiation with a material strongly depends on the absorption properties of the material. However, in contrast to infra-red radiation the MW wavelengths do not break the chemical bonds [5]. The mechanism of microwave heating is different than the conventional heating mechanism due to the propagation of heating front from the core to the surface, resulting in a larger core temperature and penetration of microwaves and depends on the sample size, frequency and power of radiation, as well as exposure time and reaction environment [5].

The quick (3 min) synthesis of exfoliated graphite materials has been prepared by 1 min microwave irradiation using different weight ratios of natural graphite and nitric acid to potassium permanganate, resulting in various expanded volume and bulk density values [7]. The graphene synthesized in about 2 h during a microwave exfoliation of GIC in deionized water resulted in a single layer thickness graphene in contrast to acetone and ethanol solvents, i.e., 3–6 nm thickness [6]. Rapid and mild thermal reduction of graphene oxide (GO) to reduced graphene (rGO) was achieved with the assistance of microwaves in a mixed solution of N,N-dimethylacetamide and water, obtaining the conductivity of about 10^4^ higher times than that of GO paper [11]. Simultaneous exfoliation and reduction of GO was obtained by treating oxide powders in a commercial microwave oven within about 1 min [12]. The microwave absorption capacity of carbon materials is dependent on their chemical composition and structure [15]. The graphene is an excellent microwave absorbent, whereas GO exhibits a poor microwave absorption capacity decreasing with increasing content of oxygen. However, the non-oxidized graphitic regions acting as a microwave absorber initiate reduction of GO under MW. The extraction of graphene and few-graphene stacks from carbon fibers was reported [18]. A mild oxidizing agent, i.e., hydrogen peroxide and additional energy source like microwave irradiation was applied for unzipping tubular carbon structures. The graphitized carbon materials of different degree of graphitization were prepared by a microwave-assisted synthesis [17]. Application of various microwave power densities and processing time provided a control of the micro-porosity, degree of graphitization influencing the electrical conductivity. The electrical, electrochemical, mechanical and other properties of GO-Pd hybrids were tailored using a microwave technique of synthesis, providing a controlled number of defects [19]. Compilation of properties of rGO prepared using a microwave assisted reduction for various power, time (from seconds to few minutes) and in different environment (air, Ar, N_2_) reported the wide range of values for C/O ratio, Raman *D* to *G* modes ratio and conductivity typical for graphene [5]. 

The effect of a microwave treatment (MWT) at 250 °C in a high pressure microwave reactor (HPMWR) at 55 bar and microwave assisted (MW at RT) conditions reduction of GO to rGO applying different reducing agents is examined. The chemical and structural properties were investigated using transmission electron microscopy (TEM), X-ray diffraction (XRD), Raman spectroscopy (Raman), infrared spectroscopy (IR) and electron spectroscopic methods like X-ray photoelectron spectroscopy (XPS), Auger electron spectroscopy (AES) and reflection electron energy loss spectroscopy (REELS). 

## 2. Materials and Methods

### 2.1. Sample Preparation

The graphene oxide, denoted as GO, was prepared from commercially available 99.0% purity graphite, denoted as Gr, from AcrosOrganics, USA, 325 mesh and expanded graphite, denoted as Gr-exp, by using a modified Hummers method [21]. These GO samples were denoted as GO-foil, GO-dried_UHV_, i.e., GO paste dried in an ultra-high vacuum (UHV) and GO-exp-foil. 

The reduction of GO was carried out in 100 mL water suspension of GO (0.6 wt%) mixed with 50 mL 1M water solution of formaldehyde (CH_2_O) and formic acid (HCO_2_H), whereas for hydrazine (N_2_H_4_) reduction with 50 mL of 50% solution of hydrazine hydrate in conditions of a microwave heating at 250 °C (MWT) in a high pressure (55 bar) microwave reactor (HPMWR) for 30 min (samples denoted as rGO-CH_2_O-MWT, rGO-HCO_2_H-MWT and rGO-N_2_H_4_-MWT). The reaction mixtures of rGO samples prepared under heating conditions were finally cooled to room temperature (RT), washed in deionized water till the pH of filtrate stabilized to ca. 7–8 and then dried.

The samples prepared under MWT conditions were compared to samples already reported elsewhere, i.e., GO and GO-exp reduced using hydrazine in 100 mL water suspension of GO (0.6 wt%) mixed with 50 mL of 50% water solution of hydrazine hydrate boiled under reflux for 30 min (rGO-N_2_H_4_, rGO-exp-N_2_H_4_), GO suspension treated for 15 min in microwaves at power at 400 W (rGO-N_2_H_4_-MW) [22], GO reduced using formaldehyde prepared in 100 mL water suspension of GO (0.6 wt%) with 50 mL 1M water solution of CH_2_O boiled under reflux for 30 min (rGO-CH_2_O), Gr and Gr-exp standards [23].

### 2.2. TEM-PEELS and XRD Equipment

A high resolution transmission electron microscopy (HR-TEM) images were measured using microscope Tecnai 20F X-Twin, equipped wth an electron source, a cathode with field emission gun (FEG), EHT = 200 keV, a camera Orius, a high-angle annular dark-field (HAADF) detector, spectrometer for energy dispersive X-ray spectroscopy (EDX) with energy resolution of 134 eV (EDAX RTEM SN9755+) and parallel electron energy loss spectroscopy (PEELS) with the energy resolution of 0.8 eV. EDX quantification was performed according to the modified standardless/thin foil method. The samples for TEM measurements were prepared by proceeding with sonication for 5 s (a few milligrams of sample in 99.8% anhydrous ethanol using ultrasound), then applying a drop (5 μL) on a carbon coated copper mesh with holes (Lacey type Cu 400 mesh, Plano) and evaporating the solvent at room temperature. Then, the remaining dried powder stuck on copper mesh was examined. 

The X-ray diffraction (XRD) spectra were recorded using an X’Pert Pro diffractometer (Cu Kα radiation. λ = 1.5406 Å, X‘Celerator detector).

### 2.3. Raman Equipment

Raman spectra were recorded using Raman micro-spectrometer (Senterra, Bruker Optik) equipped with a 532 nm laser using laser power of 2 mW, acquisition time of 360 s, number of scans 2 and in the spectra range of 800–3000 cm^−1^.

### 2.4. IR Equipment

The IR transmission spectra were recorded in a vacuum spectrometer (Vertex 70 V, Bruker Optic; T = 22 °C, *p* = 10^−1^ Pa, 70–4000 cm^−1^ range) with a resolution of 4 cm^−1^, number of scans 100, in the 750–1900 cm^−1^ range. Prior the measurements a “vacuum spectrum” was recorded to be subtracted automatically as a background from the investigated samples spectra. The samples were prepared by mixing with KBr at a ratio of 1/300 mg, then compressing at 7 MPacm^−2^ to form a pellet. 

### 2.5. XPS Equipment

The electron spectroscopic (XPS and REELS) measurements were performed in UHV chamber of the home-made ESA-31 electron spectrometer. The ESA-31 spectrometer was equipped with a hemispherical electron energy analyzer of a high energy resolution, an electron gun (LEG62-VG Microtech), a home-made X-ray excitation source (Al Kα X-rays h*ν* = 1486.67 eV) and an Ar^+^ ion source of AG21 (VG Scientific) [24]. The XPS spectra were measured in the fixed retarding ratio (FRR) mode at a photon incidence and electron emission angles of 70° and 0°, respectively, with respect to the surface normal of the specimen. The REELS spectra were recorded for a primary electrons kinetic energy of 4 keV, the electron beam current intensity of a few nA at electron incidence angle of 50° and emission angle of 0°, with respect to the specimen surface normal.

## 3. Results and Discussion

### 3.1. Structural Properties by TEM-PEELS and XRD

**Figure 1:**
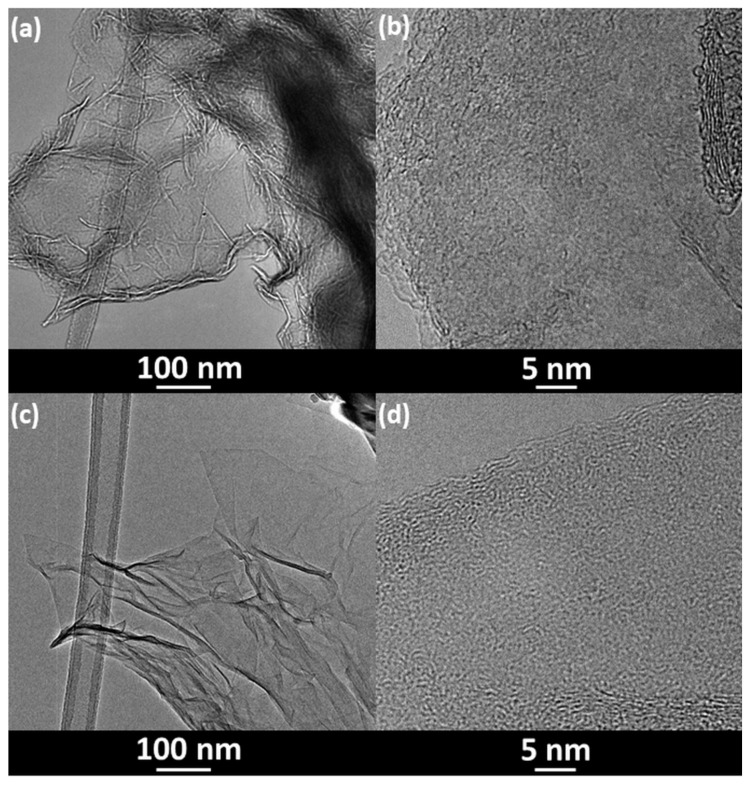
TEM images of (**a**,**b**) RGO-CH_2_O [23] and (**c**,**d**) RGO-CH_2_O-MWT.

The exemplary TEM images of rGO-CH_2_O [23] and rGO-CH_2_O-MWT indicate disordered, thin transparent structures of various transparency, where for GO-CH_2_O-MWT the diameter size of stacking nanostructures (flakes) is larger and crystallinity smaller than those for GO-CH_2_O (Figure 1).

The PEELS spectra confirm structural differences due to MWT conditions in intensity and shape of elastic and inelastic electron spectra (Figure S1—Supplementary Materials). Differences were observed in the carbon K line (C_K-edge), where for the sample rGO-CH_2_O there is a double band at 289 and 318 eV, while for the rGO-CH_2_O-MWT sample a single band at 289 eV confirming its higher structural order.

**Figure 2:**
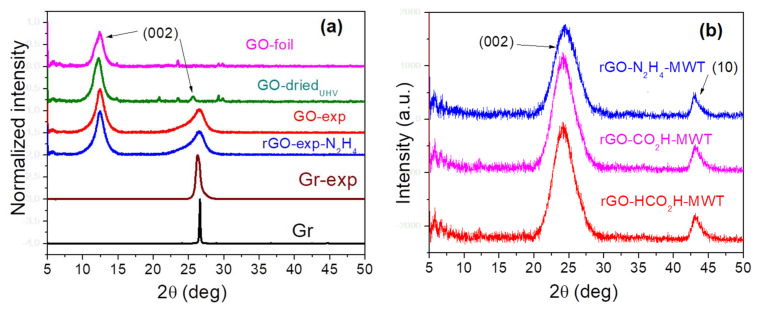
XRD patterns of: (**a**) GOs, rGO and graphites, (**b**) rGOs obtained using different reducing agents and microwave treatment (MWT).

The XRD diffractograms for the investigated samples indicating (002) and (10) reflexes, notation according to Warren [25], are shown in Figure 2. The XRD spectra were fitted using Pearson7 function after a straight-line background subtraction (Figure S2—Supplementary Materials). The parameters of the applied fitting are listed in Table S1—Supplementary Materials. The (002) reflex was applied for evaluation of graphene interlayer distance, *d*, from Bragg’s law, the average height of graphene flakes, *H*, from Scherrer’s equation (using a constant of 0.9) and then the average number of layers in graphene flakes, *n*. The (10) reflex was applied for evaluating the average diameter of graphene flakes, *D*. The evaluated parameters are shown in Table 1. For multiple (002) and (10) reflexes, the weighted average (Table S1—Supplementary Materials) values of *d*, *H* and *D* resulting from a few peaks are listed in Table 1.

The values of interlayer distance for graphene oxides vary from 0.732 nm to 0.572 nm and are the largest for GO-dried_UHV_ and the smallest for GO-exp. The remaining rGOs show values of *d* between 0.35 nm and 0.383 nm. The *d* values in rGOs obtained using the MWT procedure depend on the reducing agent, i.e., 0.35 nm (rGO-CH_2_O) and 0.375 nm (rGO-CH_2_O-MWT), whereas 0.383 (rGO-N_2_H_4_) and 0.37 (rGO-N_2_H_4_-MWT) (Table 1). Reduction procedures lead to decreasing flakes diameter of GO with different rate depending on the reducing agent, whereas the same reducing agent provides larger diameters, when using MWT procedure (Table 1).

In GOs, the average number of layers increases in the following order: GO-dried_UHV_ < GO-foil < GO-exp, whereas in rGOs in the order: 

N_2_H_4_ < CH_2_O-MWT ≈ N_2_H_4_-MWT < HCO_2_H-MWT < CH_2_O < exp-N_2_H_4_.

### 3.2. Bulk Physical and Structural Properties by Raman Spectroscopy

**Figure 3:**
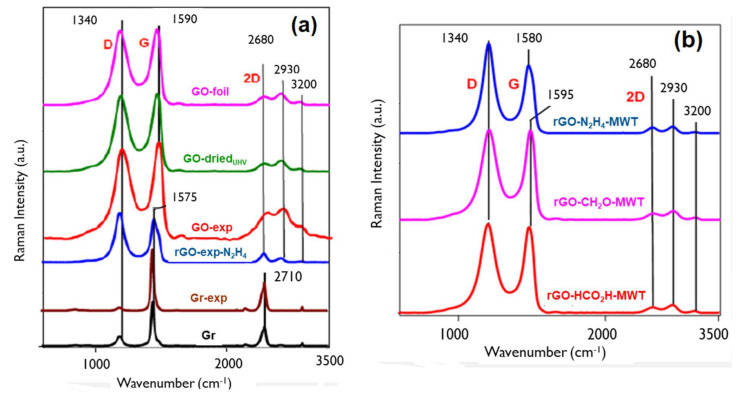
Raman spectra of: (**a**) GOs, rGO and graphites, (**b**) rGOs obtained using different reducing agents and microwave treatment (MWT).

The Raman spectra for the investigated nanomaterials show the first order modes such as *D* (~1320–1350 cm^−1^), *G* (~1570–1605 cm^−1^), *D‘* (~1620 cm^−1^), *D* + *D‘* (~2900 cm^−1^) modes and the second order 2*D* mode (2640–2680 cm^−1^) [26,27,28,29,30]. The shapes, positions, full-width at half maximum (FWHM) and dependence of FWHM on position and relative intensities are characteristic for carbon materials (Figure 3 and Figures S3–S5—Supplementary Materials; Table 2). The *D* mode occuring due to in-plane breathing vibration of carbon rings reflects disorder due to destroyed carbon hexagons present in amorphous structures. The *D* mode ntensity and FWHM is a measure of structural disorder. The *G* mode results from the in-plane stretching vibration of carbon atom pairs and is observed for all carbon structures containing sp^2^ bonds, both aromatic carbon and other sp^2^ structures. For an ideal single layer graphene, 2*D* allowed mode is the most intense, whereas for graphite a low intensity, *D* mode is accompanied by a presence of 2*D* mode. Modification of *D* and *D’* modes results from introducing defects such as (i) vacancy defects (deformation of the carbon lattice bond), (ii) on-site defects (sp^3^ hybridizations) describing atoms bonded to carbon and (iii) boundary or edge defects [31,32].

Quantification of structural parameters of carbon nanomaterials from Raman spectra, i.e., evaluation of the effective crystallite size in the direction of the graphite plane or graphitic cluster, *L_a_*, the distance between defects, *L_D_* and density of defects, *n_D_*, where *L_a_*~*L_D_* and *L_D_*~1/√*n_D_*, requires the spectra classification according to three stages amorphization trajectory [29], considering positions and FWHMs of different modes and based on (i) clustering of the sp^2^ phase, (ii) bond disorder, (iii) the presence of sp^2^ rings or chains and (iv) the sp^2^/sp^3^ ratio. Raman spectra of stage 1 (Gr, Gr-exp) exhibit a high intensity, small FWHM of *G* mode and the smallest *I*(*D*)/*I*(*G*) values (Table 3), indicating dominating graphite structure with low density of defects. The ratio of *D* to *G* mode intensities is expressed by equation:

*I*(*D*)/*I*(*G*) = *C*(*λ*)/*L_a_* (1)

where *λ* is Raman wavelength and *C* (515.5 nm) = 4.4 nm [29].

Raman spectra of amorphization trajectory stage 2 (GOs, rGOs) exhibit high intensities and FWHMs of *D* and *G* modes, very weak intensity of 2*D* mode and *I*(*D*)/*I*(*G*) values close to 1. Their characteristic features like positions, FWHM and intensity ratios of *D*, *G* and 2*D* modes (Figures S3–S5—Supplementary Materials) reveal chemical and structural differences such as thickness, C sp^3^ content, density, graphitic clusters size and crystallinity (Figure S5—Supplementary Materials, Table 3). For this stage, the new relation is valid [29]:

*I*(*D*)/*I*(*G*) = *C’*(*λ*)*L_a_*^2^ (2)

with *C’* (514 nm)~0.55 nm^−2^. The average distance between defects, *L_D_* and density of defects, *n_D_*_,_ can be also observed in *I*(*D*)/*I*(*G*) ratios since these values increase up to *L_D_*~4 nm (stage 2) and then decrease for *L_D_* > 4 nm (stage 1) [33] in agreement with a graphitization trajectory for carbon materials [29]. The FWHMs and intensities of *D* and *G* mode in GOs and rGOs indicate the distance between defects smaller than 3 nm [34]. Generally, the rGOs-MWT exhibit lower crystallinity than GOs (Figure S5—Supplementary Materials). As reported elsewhere for rGOs [29], increasing frequency of *D* mode and decreasing frequency of *G* mode accompanied by decreasing FWHM of *D* and *G* bands (Figure S3—Supplementary Materials) indicates decreasing C sp^3^ content and increasing *L_a_*. The values of *L_a_* for GO and rGO evaluated according to Equation (1) (Table 2) in the range of 1.3–1.55 nm are generally smaller for GOs. 

### 3.3. Bulk Chemical Properties by IR

**Figure 4:**
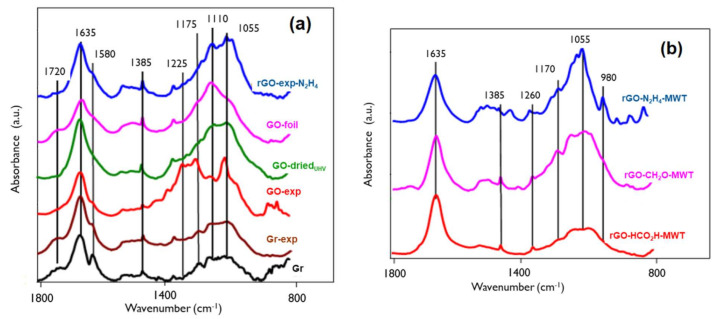
IR spectra of: (**a**) GOs, rGO and graphites, (**b**) rGOs obtained using different reducing agents and microwave treatment (MWT).

The IR spectra in the region of 800–1800 cm^−1^ are shown in Figure 4. The two regions of the absorption bands at 1750–1450 cm^−1^ and 1300–950 cm^−1^ represent C=O and C-O moieties, respectively [35,36]. The absorption band at 1720 cm^−1^ is ascribed to the stretching vibration of C=O in carboxylic (conjugated and/or non-conjugated) or carbonate systems (acid, ester, anhydride, dioxolan). The overlapping modes at 1635 cm^−1^ and 1580 cm^−1^ can be attributed to olefinic and aromatic carbon-carbon bonds (sp^2^), carbonyl moieties in various chemical surroundings (quinone-, ketone-, aldehyde-like), carbon-oxygen ion-radical structures and conjugated systems (diketone, keto-esters, keto-enol and quinone-hydroquinone structures). The absorption band at 1385 cm^–1^ is present due to aliphatic carbon-carbon bonds (sp^3^ C-H bend) [37]. At about 1630 cm^−1^, deformation vibration band *δ*(HOH) can be assigned to adsorbed/intercalated water. The overlapping absorption bands in the 1300–950 cm^−1^ region can be assigned to C-O moieties existing in different structural environment. In this spectral region, the followings can be observed: the presence of the C-O-C symmetric stretching vibration bands from epoxide group at 1290 cm^−1^, ether-, ester- lactone-, pyrone-, furane-like structure at 1225–1100 cm^–1^, C-OH vibration of phenol and hydroxyl molecular groups at 1070 cm^−1^ and alkene sp^2^ C-H bend at about 980 cm^–1^.

The spectra from graphite samples indicate the predominant contribution of absorption band from olefinic and aromatic carbon-carbon bonds (sp^2^) at 1635 cm^−1^ and 1580 cm^−1^ and C=O moieties at 1720 cm^−1^. The contribution of aliphatic carbon-carbon bonds and C-O moieties is less remarkable than in case of GO samples. 

The GO reduction procedures decrease the contribution of C=O moieties and aromatic carbon-carbon bonds, what is accompanied by increasing content of C-O moieties depending on the agent. This reduction provides different modifications in IR spectra recorded for the obtained samples. After the reduction with formaldehyde (rGO-CH_2_O) a relative decrease of the intensity of the mode attributed to carboxylic moieties (1720 cm^−1^) and a relative increase of the intensity of the mode associated with the presence of hydroxyl groups (near 1620 cm^−1^) take place [23]. Reduction using CH_2_O microwave heating assisted (rGO-CH_2_O-MWT) leads to decrease of the relative intensity of the peak ascribed to hydroxyl groups with increasing intensity of peaks in C-O-C region (carbonyl moieties, e.g., in aldehyde-like structures).

### 3.4. Evaluation of Surface Chemical and Structural Properties by REELS

**Figure 5:**
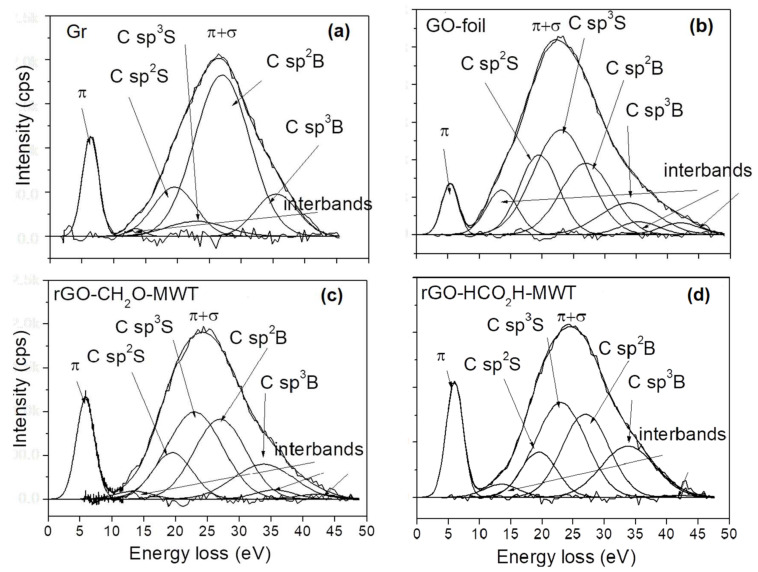
Exemplary REELS spectra fitted using Gaussian functions. Standard deviations are multiplied by 10. (**a**) Gr, (**b**) GO-foil, (**c**) rGO-CH_2_O-MWT, (**d**) rGO-HCO_2_H-MWT.

In REELS method investigates a primary electron reflected inelastically at the surface. The REELS spectrum of carbon materials reveals the chemical and structural properties of a near surface in values of intensity and energy of inelastically reflected electron losing its energy on valence electrons. For carbon the C atoms from the surface and bulk form π and π + σ bonds and exist in C sp^2^ and C sp^3^ hybridizations. The electron energy loss values were reported elsewhere for C sp^2^ containing graphite, C sp^3^containing diamond [38] and single layer graphene and graphene oxide samples [39,40]. The REELS spectra obtained from our experiments, were fitted using Gaussian functions, which reflect distribution of velocities leading to Doppler broadening. The energy loss values for bulk (B) and surface (S) C sp^2^ and C sp^3^ components, denoted as C sp^2^B, C sp^2^S, C sp^3^B, C sp^3^S, as reported previously for graphite, diamond [38] and graphite nanomaterials [22,23] were applied (Figure 5 and Figure S6—Supplementary Materials). Similarly, the FWHMs of the respective Gaussian components were applied, as shown previously for graphite and diamond [38]. 

The values of intensity and electron energy loss of all components are presented in Table 3. For a one-layer graphene due to non-existing components from bulk atoms, the intensity ratio of C sp^2^B to C sp^2^S is zero and then increases with the increasing number of layers due to contribution from bulk atoms present in further layers. For graphite, this intensity ratio is determined as a number of layers, depending on electron information depth related with measurement geometry and electron kinetic energy [41,42]. Assuming an exponential interpolation of C sp^2^B/C sp^2^S intensity ratio between graphene and graphite, where the backscattered electron travelling through a thickness *z* with an inelastic mean free path value, *λ*, is emitted in angle with respect to the surface normal, *α*_out_, loses its intensity, *I*, where *I* = *I*^∞^ (1 − exp(−*z*/*λ*cos*α*_out_) [43], the values of thickness and then the number of layers in samples prepared using different chemical procedures can be evaluated [22]. When determining the number of layers, *n*, from REELS the values of interlayer distance, *d*, evaluated from XRD (Table 1) were considered. The values of *n* resulting from REELS and averaged values resulting from REELS and XRD (Table 1) are listed in Table 3. The values of average number of layers in flakes obtained from XRD (Table 1) and REELS (Table 3) are consistent.

The GOs show the least number of flake layers, i.e., GO-dried_UHV_ < GO-foil < GO-exp. The rGOs resulting from MWT exhibit the smallest number of flake layers for reducing conditions: CH_2_O-MWT < HCO_2_H-MWT < N_2_H_4_-MWT. The N_2_H_4_ reducing agent accompanied by MW and MWT procedures leads to increasing number of flake layers with no remarkable difference between MW and MWT treatments. In contrast, the CH_2_O reducing agent MWT procedure provides smaller number of flake layers in comparison to only CH_2_O reducing agent. Reduction using N_2_H_4_ of GO-exp provides larger number of flake layers in comparison to reduction of GO prepared from graphite.

### 3.5. Surface Elemental Content by XPS

**Figure 6:**
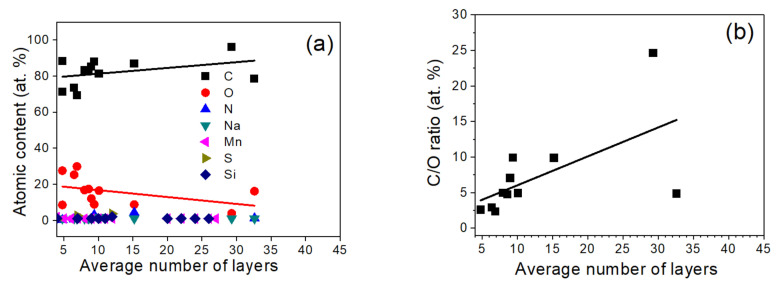
XPS determined: (**a**) surface atomic content (**b**) ratio of C to O atomic content dependent on the average number of layers in the investigated samples.

XPS analyzes the intensity and binding energy (BE) values of photoelectrons from different atoms and their orbitals, where the intensity and BE are attributed to surface atomic concentration and chemical state of atom at the surface, respectively. The atomic content of elements present at the surface (C, O, N and contamination such as Na, Si, S and Mn) was quantified from the survey XPS spectra accounting for an area under C 1s, O 1s, N, 1s, Na 1s, Si 2p, S 2p and Mn 2p photoelectron peaks after Tougaard background subtraction [44] applying XPS MultiQuant software [45,46], using Scofield photoionization cross-sections [47] and corrections for analyzer transmission function and electron inelastic scattering. The investigated samples show at the surface predominantly the presence of C, O and traces of S, N, Mn, Si and Na (Table S2—Supplementary Materials and Figure 6). The contaminations of S and N results from sulfuric and nitric acid used during GO and/or expanded graphite preparation. The GOs show a higher content of O in comparison to rGOs, expanded graphite and graphite (Table S2—Supplementary Materials). The content of C increases and content of O decreases with increasing number of flake layers (Figure 6a), which is accompanied by increasing C/O ratio (Figure 6b).

### 3.6. Surface Content of Carbon Hybridizations and Oxygen Groups by XPS and AES

The carbon and oxygen chemical forms and their content were investigated from fitting of C 1s and O 1s spectra after Tougaard background subtraction [44] using XPSPeakfit software [48]. For fitting the XPS spectra, a mixture of Lorentzian and Gaussian (30:70) was applied since Lorentz and Gaussian describe distribution of decaying oscillations and velocities, respectively. 

**Figure 7:**
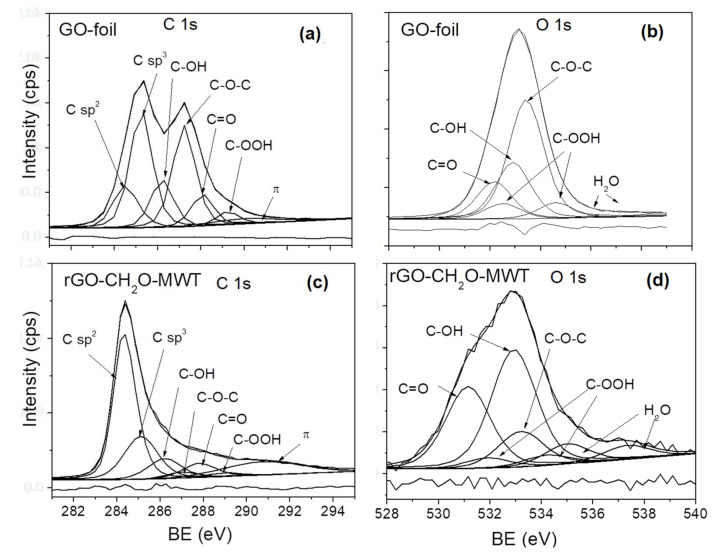
The C 1s and O 1s XPS spectra fitting using asymmetric Gaussian-Lorentzian function for the selected GOs and rGOs. (**a**,**b**) GO-foil, (**c**,**d**) rGO-CH_2_O-MWT.

The following chemical forms of C atom in C 1s spectrum were assumed in the fitting: C sp^2^ bonds, vacancy and C sp^3^ defects and carbon oxygen groups like hydroxyl (C-OH), epoxy (C-O-C), carbonyl (C=O) and carboxyl (C-OOH), whereas in O 1s spectra fitting, C-OH, C-O-C, C=O, C-OOH and adsorbed water. The values of BE in the fitting of C 1s and O 1s spectra were considered according to the literature data [49,50,51,52,53,54,55]. The FWHM ratios of different carbon chemical states in C 1s spectra were applied according to Ref. [54], whereas for O 1s similar values were assumed. The C 1s and O 1s spectra are presented in Figure 7, Figures S7 and S8—Supplementary Materials, whereas the atomic content of C and O chemical states is listed in Tables S3 and S4—Supplementary Materials.

The differences between GO prepared from Gr and GO-exp and GO dried in vacuum are observed in content of O, i.e., GO-exp > GO-foil > GO-dried_UHV_ (Table S2—Supplementary Materials), carbon oxygen groups, i.e., GO-foil > GO-dried_UHV_ > GO-exp (Table S3—Supplementary Materials), vacancy and C sp^3^ defects, i.e., GO-dried_UHV_ > GO-foil > GO-exp (Table S3—Supplementary Materials) and adsorbed water, i.e., GO-exp > GO-foil > GO-dried_UHV_ (Table S4—Supplementary Materials), which justifies the oxygen content at the surface resulting from adsorbed water. The average number of layers in GO flakes increases in the following order: GO-dried_UHV_ < GO-foil < GO-exp (Table 3), where the interlayer distance in GO-dried_UHV_≈GO-foil is larger than in GO-exp (Table 1).

**Figure 8:**
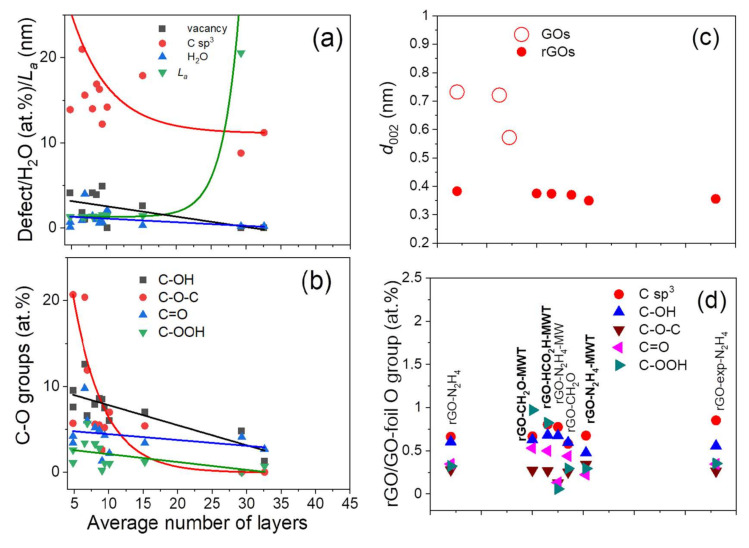

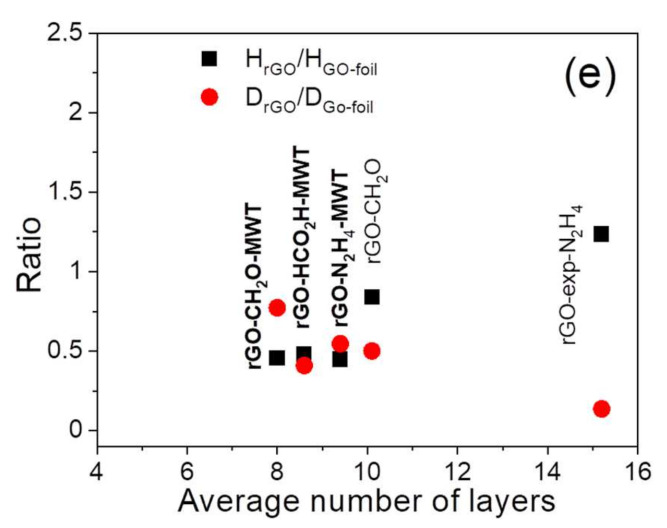
(**a**) Surface atomic content of vacancy, C sp^3^ defects, adsorbed H_2_O and effective crystallite size in direction of graphite plane *L_a_*, (**b**) carbon-oxygen groups content, (**c**) distance between graphene layers, *d*_002_, (**d**) ratio of rGO to GO-foil oxygen group content determined from C 1s spectra fitting (Table S2), (**e**) ratio rGO to GO-foil flakes height and diameter resulting from XRD (Table 1) as a function of the average number of layers.

The number of flake layers increases with decreasing content of vacancy, C sp^3^ defects, oxygen groups and adsorbed water leading to increasing *L_a_* (Figure 6a,b), what is accompanied by decreasing interlayer distance (Table 1, Figure 8c). In GOs, the water between the interstitial graphene layers bonded to a large amount of oxygen groups through hydrogen bonding results in a larger interlayer distance. Reduction removes carbon oxygen groups and water, which may remain as adsorbed at the surface, leading to decreasing interlayer distance. For rGOs with increasing flake layers, the highest decrease rate is observed for C-O-C groups and then C-OH groups, whereas C=O > C-OOH groups decrease at a slower rate (Figure 6b). The MWT treatment using CH_2_O provides slower decrease of C-OOH groups in GO in comparison to no MWT reductions (Figure 8d). Simultaneously, the MWT treatment at 250 °C increases vacancy defects, decreases C sp^3^ defects (Figure 8a) and does not modify strongly the diameter of GO (Figure 8e). This would suggest that MWT facilitates formation of C-OH, C=O and C-OOH groups at vacancies and provides more competitive penetration of reducing agent between the interstitial sites than via vacancies. These additional oxygen groups may result from water dissociation and/or removal of oxygen from epoxy group due to using MWT conditions. The smaller number of flake layers is shown for rGOs, where reduction of GOs oxygen groups (Figure 8e) between the interstitial sites is faster than those in the flake planes. These processes depend on the reducing conditions, i.e., reducing agent and its ability to interact with oxygen and hydrogen atoms in oxygen groups and water, reducing agent kinetics, dynamic interaction of MW with the material treated and the obtained temperature due to MW treatment, which depend on absorption properties of the treated material since the thermal decomposition of oxygen groups is temperature dependent [56,57].

Evaluation of the content of C sp^2^/sp^3^ bonds utilizing different surface sensitivity of XPS, REELS and AES spectra has been performed previously [23,49]. A measure of surface sensitivity is the information depth (*ID*) [41,42]. The average information depth, i.e., a mean escape depth (*MED*) in XPS and AES and mean penetration depth (*MPD*) in REELS, are defined as an average depth normal to the surface from which the specified particles escape and for assumed single scattering of electrons can be evaluated from equations reported previously [41,42]. Accounting for the values of electron inelastic mean free path for graphite from Shinotsuka et al. [58] and applied geometry of analysis the following values can be obtained: REELS-bulk (4000 eV)—10 nm (*ID* (99%), 2.17 nm (*MPD*), REELS-bulk (2000 eV)—6.53 nm (*ID* (99%), 1.42 nm (*MPD*), C 1s-EELS XPS—9.72 nm (*ID* (99%), 2.11 nm (*MED*) and C KLL AES—3.32 nm (*ID* (99%), 0.72 nm (*MED*). Therefore, *ID* increases in the following order:

REELS-surface < C KLL AES < REELS-bulk (2000 eV) < REELS-bulk (4000 eV) ≈ C 1s-EELS XPS, where the REELS-surface component provides information from the first outer layer of graphene. The percent contribution of REELS bulk and surface components of C sp^2^/sp^3^ hybridized atoms are listed in Table 3, whereas the C sp^2^/sp^3^ contributions evaluated from C 1s spectra in Table S3—Supplementary Materials. The C sp^3^ content at the surface sensitivity of C KLL spectra is evaluated from parameter D, i.e., the energy distance between maximum and minimum intensity values of the first derivative of C KLL spectra, (Figure S9—Supplementary Materials, Table 4), assuming a linear interpolation of parameter D for C sp^2^ (graphite) and C sp^3^ (diamond) standards from [49] as proposed by Lascovic et al. [59].

**Figure 9:**
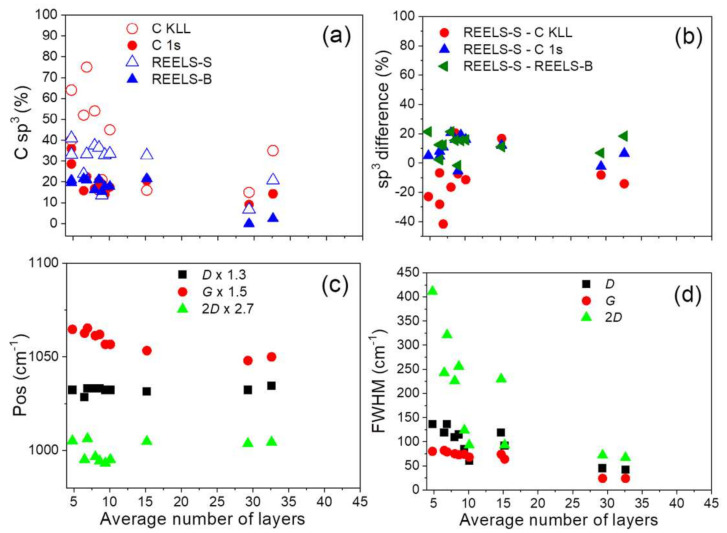
(**a**) C sp^3^ content determined from C KLL AES spectra and parameter D (Table 4), C 1s spectra fitting (Table S2) and REELS-surface (REELS-S) and REELS-bulk (REELS-bulk) components (Table 3), (**b**) the respective differences, Raman spectra *D*, *G*, 2*D* modes (**c**) positions (Table 2) and (**d**) FWHMs (Table 2) dependent on the average number of layers in the investigated samples.

The C sp^3^ content at various surface information depths is compared in Figure 9a,b. The *MED* for C 1s-EELS XPS and REELS-bulk (4000 eV) is about 3–7 graphene layers, for C KLL spectra—1–2 graphene layers, respectively, whereas REELS-surface component reflects information from the outer layer. Discrepancies between C sp^3^ for first graphene layers (REELS-surface component and C KLL AES) and further layers (C 1s-EELS XPS and REELS-bulk component) result from non-homogeneous distribution of C sp^3^ and oxygen groups of the largest content at the flakes plane (Figure 9a, Table 4). Otherwise, C sp^3^ content resulting from C 1s-EELS XPS and REELS-bulk is similar nearly for all the samples. For rGOs with larger number of flake layers, the content of C sp^3^ decreases indicating smaller number of C sp^3^ defects and oxygen groups. The difference between C sp^3^ hybridizations resulting from REELS-surface and C KLL spectra provides proportional dependence with increasing number of layers in contrast to the decreasing respective difference resulting from REELS-surface and REELS-bulk (and REELS-surface and C 1s) analyses (Figure 9b). This means that reduction of oxygen groups at the flakes plane and between the layers proceed with different rates.

The reduction accompanied by MWT using N_2_H_4_ and CH_2_O provides larger content of vacancy, C sp^3^ defects and carbon oxygen groups (Table S3—Supplementary Materials, Figure 9a,b) due to probably interstitial water dissociation and/or removal of oxygen from epoxy groups providing additional functionalization and various in-depth distribution of C sp^3^ bonds (Figure 9a,b). The MW and MWT conditions provide an additional kinetic and thermal energy for a reducing agent penetration between the interlayers and heating the sample, where the temperature will vary depending on the absorption properties of material. For N_2_H_4_ MW and MWT accompanied reduction, in comparison to N_2_H_4_ reduction, a slight increase of the number of layers is obtained, whereas the CH_2_O MWT reducing procedure decreases the number of layers in comparison to a procedure using only CH_2_O (Table 3). The structural and chemical properties are confirmed in Raman spectra features (Figure 9c,d and S10—Supplementary Materials), where dependence of number of flake layers and vacancy and C sp^3^ defects on Raman *G*, *D* and 2*D* modes positions, FWHMs and intensity ratios are observed. The increasing number of layers in graphene flakes related to decreasing number of C sp^3^ and vacancy defects (Figure S10—Supplementary Materials) is manifested in decreasing position of 2*D* and *G* bands (Figure 9c) and decreasing FWHM of 2*D* > *D* > *G* bands (Figure 9d).

## 4. Conclusions

The application of experimental methods of analysis such as TEM, XRD, Raman, IR, XPS/AES and REELS revealed differences in the chemical and structural properties of: (i) GO samples prepared using a modified Hummers method (graphite and expanded graphite, GO prepared from graphite dried in the atmosphere and UHV) and (ii) rGOs prepared in aqueous solutions of N_2_H_4_, CH_2_O and HCO_2_H accompanied by a microwave heating at 250 °C in a high pressure microwave reactor at 55 bar. For comparison, the rGOs reduced using N_2_H_4_, N_2_H_4_ microwave assisted and CH_2_O were considered. 

Samples showed different interlayer distance, flake diameter, number of vacancy and C sp^3^ defect, oxygen groups and adsorbed water. The GO dried in UHV resulted in a smaller number of layers, larger number of vacancy and C sp^3^ defects and a smaller number of oxygen groups (except C-O-C) and adsorbed water than GO dried in atmosphere. GO-exp and GO-foil show larger number of flake layers, smaller number of defects and larger content of adsorbed water than GO-dried_UHV_. This would suggest that in UHV the interstitial water and weakly bonded C-OH, C-OOH and C=O groups are being removed, creating more defects than in GO-foil dried in the atmosphere, where water can dissociate forming additional oxygen groups. 

The MWT conditions result in formation of larger amount of vacancy defects and in the case of weaker reducing agent like CH_2_O, lead to increasing content of oxygen groups, resulting probably from water dissociation and/or removal of oxygen from epoxy group, leading to a smaller number of flake layers. In the case of a strong N_2_H_4_ reducing agent accompanied by microwaves (MH, MWT), the content of oxygen groups is not modified due to microwave treatment; however, these procedures result in a larger number of flake layers. The rGOs prepared using MWT procedure showed the average number of layers in the following order: CH_2_O-MWT < HCO_2_H-MWT < N_2_H_4_-MWT. Number of layers in rGOs increases with decreasing content of vacancy, C sp^3^ defects, oxygen groups, water and flake diameter. This would suggest that MWT facilitates formation of oxygen groups at vacancies and provides more competitive penetration of reducing agent between the interstitial sites than via vacancies. The smaller number of flake layers show rGOs, where reduction of GOs oxygen groups between the interstitial sites is faster than those in the flake planes. This process depends on reducing agent, its reducing ability and dynamic interaction of MWT conditions with the treated material. Different reducing agents at MWT conditions resulted in various in-depth distribution of C sp^3^ at the surface and in the bulk, what depends on MW kinetic and thermal effect on the treated material modifying the rates of oxygen group decomposition.

The microwave assisted methods of GO reduction, being more efficient, less energy and time consuming, may be utilized for a large scale graphene production.

## Figures and Tables

**Table 1:** GOs and rGOs parameters obtained from XRD patterns, i.e., average distance between graphene layers (*d*), average height of flakes (*H*), average number of graphene layers (*n*) and average diameter of flakes (*D*).

Sample	Peak (002)			Peak (10)
	Average *d* (nm)	Average *H* (nm)	Average *n*	Average *D* (nm)
* GO-foil	0.721	4.883	6.8	* 22
GO-dried_UHV_	0.732	4.625	6.3	-
* rGO-N_2_H_4_	0.383	1.302	3.4	8
rGO-N_2_H_4_-MWT	0.370	2.188	5.9	12
GO-exp	0.572	4.288	7.5	-
rGO-exp-N_2_H_4_	0.356	6.028	16.9	3
* rGO-CH_2_O	0.350	4.099	11.7	11
rGO-CH_2_O-MWT	0.375	2.217	5.9	17
rGO-HCO_2_H-MWT	0.374	2.349	6.3	9
* Gr	0.344	-	-	-
* Gr-exp	0.347	-	-	-

* from [23].

**Table 2:** The position, full-width at half maximum (FWHM) values and intensity ratios of *D*, *G* and 2*D* modes of Raman spectra recorded from graphite, GOs and rGOs. and the effective crystallite size in direction of graphite plane (*L_a_*).

Sample	*D* Band		*G* Band		2*D* Band		*I*(*D*)/ *I*(*G*)	*I*(2*D*)/ *I*(*G*)	*L_a_* (nm)
	Pos (cm^−1^)	FWHM (cm^−1^)	Pos (cm^−1^)	FWHM (cm^−1^)	Pos (cm^−1^)	FWHM (cm^−1^)			
* GO-foil	1337	119	1594	82	2687	243	0.991	0.465	1.34
GO-dried_UHV_	1342	136	1597	80	2714	411	0.927	0.2611	1.30
rGO-N_2_H_4_-MWT	1342	85	1585	74	2682	124	1.32	0.0833	1.55
GO-exp	1343	136	1598	79	2717	321	0.928	0.2632	1.30
rGO-exp-N_2_H_4_	1341	92	1580	64	2713	93	1.139	0.2033	1.44
* rGO-CH_2_O	1342	60	1585	68	2687	93	1.242	0.7210	1.5
rGO-CH_2_O-MWT	1343	109	1592	75	2691	226	1.036	0.0635	1.37
rGO-HCO_2_H-MWT	1343	115	1593	73	2685	256	1.002	0.5884	1.35
* Gr	1342	45	1572	24	2710	72	0.214	0.4315	20.56
* Gr-exp	1345	42	1575	24	2712	67	0.041	0.4604	107.32

* from [23].

**Table 3:** REELS spectral parameters evaluated for the investigated carbon nanomaterials.

Sample	π + σ Contributions (%)					Average Number of Layers, *n*	
	C sp^2^S E_loss_ = 19.5 eV	C sp^2^B E_loss_ = 27 eV	C sp^3^S E_loss_ = 23 eV	Csp^3^B E_loss_ = 33.8 eV	Csp^2^B/Csp^2^S	*d* from XRD (Table 1)	Average Value of *n* (XRD and REELS)
* GO-foil	13.0	33.0	33.2	20.8	2.54	6.1	6.5 ± 0.4
GO-dried_UHV_	15.9	23.4	41.0	19.7	1.47	3.2	4.8 ± 2.2
** rGO-N_2_H_4_	15.6	39.0	23.8	21.6	2.51	6.1	4.8 ± 1.9
** rGO-N_2_H_4_ -MW	10.6	34.5	13.6	15.3	3.26	9.0	9.0
rGO-N_2_H_4_-MWT	13.4	35.9	33.0	17.7	2.67	12.8	9.4 ± 4.9
GO-exp	14.5	31.2	33.3	21.0	2.15	6.2	6.9 ± 0.9
rGO-exp-N_2_H_4_	12.4	33.3	32.7	21.6	2.69	13.4	15.2 ± 2.5
* rGO-CH_2_O	17.2	31.4	33.6	17.8	1.83	8.4	10.1 ± 2.3
rGO-CH_2_O-MWT	14.3	32.0	37.5	16.2	2.24	10.0	8.0 ± 2.9
rGO-HCO_2_H-MWT	12.5	29.8	36.5	21.1	2.39	10.9	8.6 ± 3.3
* Gr	18.5	74.7	6.8	0	4.04		29.3
* Gr-exp	14.7	61.9	20.8	2.5	4.21		32.6

* from [23], ** [22].

**Table 4:** Values of parameter D determined from the first derivative of C KLL AES spectra, C sp^3^ percent resulting from parameter D and fitting of C 1s spectra. Evaluation of C sp^3^ from C KLL spectra was performed using a calibration curve and parameter D for standards of 13.2 eV (C sp^3^) and 23.1 eV (C sp^2^) [49].

Sample	Parameter D (eV)	C sp^3^ (%) (C KLL)	C sp^3^ (%) (C 1s)
* GO-foil	19.1	40	28.6
GO-dried_UHV_	16.8	64	36
** rGO-N_2_H_4_	18.0	52	15.8
** rGO-N_2_H_4_-MW	21.0	21	19.1
rGO-N_2_H_4_-MWT	21.5	16	13.9
GO-exp	15.7	75	22.5
rGO-exp-N_2_H_4_	21.5	16	20.6
* rGO-CH_2_O	18.7	45	17.5
rGO-CH_2_O-MWT	17.8	54	16.8
rGO-HCO_2_H-MWT	21.5	16	20.5
* Gr	21.6	15	9.2
* Gr-exp	19.6	35	14.3

* from [22], ** from [23].

## Data Availability

The data presented in this study are available in Supplementary Information.

## Supplementary Materials

The following are available online at https://www.mdpi.com/article/10.3390/ma14195728/s1. Figure S1: PEELS spectra of (**a**) rGO-CH_2_O [23] and (**b**) rGO-CH_2_O-MWT. Figure S2: Results of XRD spectra fitting using Pearson7 function for the investigated samples, Table S1: Parameters of XRD spectra recorded from the investigated samples. These parameters were considered in evaluation of structural properties of GO and rGO like an interlayer distance, *d*, average number of stacking nanostructures, *H*, average number of layers in stacking nanostructurs, *n*, and average diameter of stacking nanostructures, *D*, (Table 1), Figure S3: The values of position and FWHM of Raman spectra recorded from the investigated samples indicated from top to bottom in the order starting from GO/rGOs to graphite, Figure S4: Dependence of Raman *D*, *G* and 2*D* band position on the FWHM in the investigated samples indicated from top to bottom, Figure S5: The ratio of intensities of 2*D* to *D* band for the investigated samples indicated from top to bottom in the order of increasing crystallinity, Figure S6: Results of REELS spectra fitting using Gaussian functions. Standard deviation multiplied by 10, Table S2: Surface atomic concentration of nanocarbon materials determined using XPS, Figure S7: Results of C 1s spectra fitting using asymmetric Gaussian-Lorentzian functions, Figure S8: Results of O 1s spectra fitting using Gaussian-Lorentzian functions, Table S3: Atomic content of C sp2/sp^3^ hydridizations and carbon-oxygen groups resulting from C 1s spectra fitting using Gaussian-Lorentzian asymmetric functions, Table S4: Atomic content of carbon-oxygen groups and water resulting from O 1s spectra fitting using Gaussian-Lorentzian asymmetric functions, Figure S9: Exemplary first derivative C KLL Auger spectra (smoothing using weighted average with 9 window points) recorded for carbon nanomaterials for evaluation of parameter D, Figure S10: Dependence of C sp^3^ and vacancy defects on position, FWHM and intensity of Raman spectra for the investigated samples.

## References

[1] Zhuo Q., Mao Y., Lu S., Cui B., Yu L., Tang J., Sun J., Yan C. (2019). Seed-assisted synthesis of graphene films on insulating substrate. Materials.

[2] Kamedulski P., Ilnicka A., Lukaszewicz J.P. (2018). Selected aspects of graphene exfoliation as an introductory step towards 3D structuring of graphene nano-sheets. Curr. Graphene Sci..

[3] Prekodravac J.R., Kepić D.P., Colmenares J.C., Giannakoudakis D.A., Jovanović S.P. (2021). A comprehensive review on selected graphene synthesis methods: From electrochemical exfoliation through rapid thermal annealing towards biomass pyrolysis. J. Mater. Chem. C.

[4] Jakhar R., Yap J.E., Joshi R. (2020). Microwave reduction of graphene oxide. Carbon.

[5] Xie X., Zhou Y., Huang K. (2019). Advances in microwave-assisted production of reduced graphene oxide. Front. Chem..

[6] Kim H.-R., Lee S.-H., Lee K.-H. (2018). Scalable production of large single-layers graphenes by microwave exfoliation in deionized water. Carbon.

[7] Wei T., Fan Z., Luo G., Zheng C., Xie D. (2008). A rapid and efficient method to prepare exfoliated graphite by microwave irradiation. Carbon.

[8] Falcao E.H.L., Blair R.G., Mack J.J., Viculis L.M., Kwon C., Bendikov M., Kaner R.B., Dunn B.S., Wudl F. (2007). Microwave exfoliation of a graphite intercalation compound. Carbon.

[9] Sridhar V., Jeon J.-H., Oh I.-K. (2010). Synthesis of graphene nano-sheets using eco-friendly chemicals and microwave ratioation. Carbon.

[10] Tryba B., Morawski A.W., Inagaki M. (2005). Preparation of exfoliated graphite by microwave irradiation. Carbon.

[11] Chen W., Yan L., Bangal P.R. (2010). Preparation of graphene by the rapid and mild thermal reduction of graphene oxide induced by microwaves. Carbon.

[12] Zhu Y., Murali S., Stoller M.D., Velamakanni A., Piner R.D., Ruoff R.S. (2010). Microwave assisted exfoliation and reduction of graphite oxide for ultracapacitors. Carbon.

[13] Yang J., Jo M.R., Kang M., Huh Y.S., Jung H., Kang Y.-M. (2014). Rapid and controllable synthesis of nitrogen doped reduced graphene using microwave-assisted hydrothermal reaction for high power-density supercapacitors. Carbon.

[14] Zhao Y., He J. (2019). Novel template-assisted microwave conversion of graphene oxide to graphene patterns: A reduction transfer mechanism. Carbon.

[15] Hu H., Zhao Z., Zhou Q., Gogotsi Y., Qiu J. (2012). The role of microwave absorption on formation of graphene from graphite oxide. Carbon.

[16] Quan B., Liang X., Ji G., Lv J., Dai S.S., Xu G., Du Y. (2018). Laminated graphene oxide-supported high-efficiency microwave absorber fabricated by an in situ growth approach. Carbon.

[17] Canal-Rodríques M., Arenillas A., Menéndez J.A., Beneroso D., Rey-Raap N. (2018). Carbon xerogels graphitised by microwave heating as anode materials in lithium-ion batteries. Carbon.

[18] Sridhar V., Jeon J.-H., Oh I.-K. (2011). Microwave extraction of graphene from carbon fibres. Carbon.

[19] Kumar R., Savu R., Singh R.K., Joanni E., Singh D.P., Tiwari V.S., Vaz A.R., da Silva E.T.S.G., Maluta J.R., Kubota L.T. (2017). Controlled density of defects assisted perforated structure in reduced graphene oxide nanosheets-palladium hybrids for enhanced ethanol electro-oxidation. Carbon.

[20] Kuang B., Song W., Ning M., Li J., Zhao Z., Guo D., Cao M., Jin H. (2018). Chemical reduction dependent dielectric properties and dielectric loss mechanism of reduced graphene oxide. Carbon.

[21] Hummers W.S., Offeman R.E. (1958). Preparation of graphitic oxide. J. Am. Chem. Soc..

[22] Stobinski L., Lesiak B., Malolepszy A., Mazurkiewicz M., Mierzwa B., Zemek J., Jiricek P., Bieloshapka I. (2014). Graphene oxide and reduced graphene oxide studied by the XRD, TEM and electron spectroscopy methods. J. Electron Spectrosc. Rel. Phenom..

[23] Lesiak B., Trykowski G., Tóth J., Biniak S., Kövér L., Rangam N., Stobinski L., Malolepszy A. (2021). Chemical and structural properties of reduced graphene oxide–dependence on the reducing agent. J. Mat. Sci..

[24] Kövér L., Varga D., Cserny I., Tóth J., Tőkési J. (1992). Some applications of high-energy, high-resolution Auger-electron spectroscopy using Bremsstrahlung radiation. Surf. Interface Anal..

[25] Warren B.E. (1941). X-Ray Di8raction in Random Layer Lattices. Phys. Rev..

[26] Malard L.M., Pimenta M.A., Dresselhaus G., Dresselhaus M.S. (2019). Raman spectroscopy in graphene. Phys. Rep..

[27] Qi Z., Zhu X., Jin H., Zhang T., Kong X., Ruoff R.S., Qiao Z., Ji H. (2018). Rapid identification of the layer number of large-area-graphene on copper. Chem. Matter..

[28] Szirmai P., Márkus B.G., Chacón-Torres J.C., Eckerlein P., Edelthalhammer K., Englert J.M., Mundloch U., Hirsch A., Hauke F., Náfrádi B. (2019). Characterizing the maximum number of layers in chemically exfoliated graphene. Sci. Rep..

[29] Ferrari A.C., Robertson J. (2000). Interpretation of Raman spectra of disordered and amorphous carbon. Phys. Rev. B.

[30] Tunistra F., Koenig J.L. (1970). Raman spectrum of graphite. J. Chem. Phys..

[31] Casiraghi C., Pisana S., Novoselov K.S., Geim A.K., Ferrari A.C. (2007). Raman fingerprint of charged impurities in graphene. Appl. Phys. Lett..

[32] Eckmann A., Felten A., Mishchenko A., Britnell L., Krupke R., Novoselov K.S., Casiraghi C. (2012). Probing nature of defects in graphene by Raman spectroscopy. Nano Lett..

[33] Lucchese M.M., Stavale F., Martin Ferreire E.H., Vilani C., Moutinho M.V.O., Capaz R.B., Achete C.A., Jorio A. (2010). Quantifying ion-induced defects and Raman relaxation length in graphene. Carbon.

[34] Cançado L.G., Jorio A., Martins Ferreira E.H., Sravale F., Achete C.A., Capaz R.B., Moutinho M.V.O., Lombardo A., Kulmala T.S., Ferrari A.C. (2011). Quantifying defects in graphene via Raman Spectroscopy at different excitation energies. Nano Lett..

[35] Ren P.-G., Yan D.-X., Ji X., Che T., Li Z.-M. (2011). Temperature dependence of graphene oxide reduced by hydrazine hydrate. Nanotechnology.

[36] Guo H., Peng M., Zhu Z., Sun L. (2013). Preparation of reduced graphene oxide by infrared irradiation induced photothermal reduction. Nanoscale.

[37] Robertson J. (2002). Diamond-like amorphous carbon. Mat. Sci. Eng. R.

[38] Calliari L., Fanczenko S., Filippi M. (2007). Plasmon features in electron energy loss spectra from carbon materials. Carbon.

[39] Eberlein J.T., Bangert U., Nair R.R., Jones R., Gass M., Bleloch A.L., Novoselov K.S., Geim A., Briddon P.R. (2008). Plasmon spectroscopy of free-standing graphene films. Phys. Rev. B Condens. Matter..

[40] Johari P., Shenoy V.B. (2011). Modulating optical properties of graphene oxide: Role of prominent functional groups. ACS Nano.

[41] Jablonski A., Powell C.J. (2004). Information depth for elastic-peak electron spectroscopy. Surf. Sci..

[42] Jablonski A., Powell C.J. (2009). Practical expressions for the mean escape depth, the information depth, and the effective attenuation length in Auger-electron spectroscopy and x-ray photoelectron spectroscopy. J. Vac. Sci. Technol. A.

[43] Jablonski A., Zemek J. (2009). Overlayer thickness determination by XPS using the multiline approach. Surf. Interface Anal..

[44] Touggard S. Background Analysis of XPS/AES QUASES Simple BackgroundsVer. 2.2, 1999–2001 Tougaard Inc. http://www.quases.com.

[45] Mohai M. (2004). XPS MultiQuant: Multimodel XPS quantification software. Surf. Interface Anal..

[46] Mohai M. Multimodel of X-ray photoelectron spectroscopy qunatification program for 32-bit Windows, XPSMuliQuant, ver. 7. 1999–2001.

[47] Scofield H. (1976). Hartree-Slater Subshell Photoionization Cross-sections at 1254 and 1487 eV. J. Electron Spectrosc. Relat. Phenom..

[48] Kwok R.W.M. XPS Peak Fitting Program for WIN95/98 XPSPEAK, ver. 4.1, Department of Chemistry, The Chinese University of Hong Kong, rmkwok@cuhd.edu.hk. http://www.uksaf.org/software.html>XPSPEAK4.1.

[49] Lesiak B., Kövér L., Tóth J., Zemek J., Jiricek P., Kromka A., Rangam N. (2018). C sp2/sp3 hybridisations in carbon nanomaterials–XPS and (X) AES study. Appl. Surf. Sci..

[50] Butenko Y.V., Krishnamurthy S., Chakraborty A.K., Kuznetsov V.L., Dhanak V.R., Hunt M.C., Šiller L. (2005). Photoemission study of onionlike carbons produced by annealing nanodiamonds. Phys. Rev. B.

[51] Shim S.H., Kim K.T., Lee J.U., Jo W.H. (2012). Facile method to functionalize graphene oxide and its application to poly (ethylene terephthalate)/graphene composite. ACS Appl. Mater. Interfaces.

[52] Fujimoto A., Yamada Y., Koinuma M., Sata S. (2016). Origins of sp3C peaks in C1s X-ray photoelectron spectra of carbon materials. Anal. Chem..

[53] Winter B., Aziz E.F., Hergenhahn U., Faubel M., Hertel I.V. (2007). Hydrogen bonds in liquid water studied by electron spectroscopy. J. Chem. Phys..

[54] Yamamoto S., Bluhm H., Andersson K., Ketteler G., Ogasawara H., Salmeron M., Nilsson A. (2008). In situ x-ray photoelectron spectroscopy studies of water on metals and oxides at ambient conditions. J. Phys. Condend. Matter..

[55] Estrade-Szwarckopf H. (2004). XPS photoemission in carbonaceous materials: A ‘‘defect’’ peak beside the graphitic asymmetric peak. Carbon.

[56] Stobinski L., Lesiak B., Zemek J., Jiricek P., Biniak S., Trykowski G. (2010). Studies of oxidized carbon nanotubes in temperature range RT-630oC by the infrared and electron spectroscopies. J. Alloy. Compd..

[57] Lesiak B., Zemek J., Jiricek P., Stobinski L. (2009). Temperature modification of oxidized multiwall carbon nantubes studied by electron spectroscopy methods. Phys. Status Solidi.

[58] Shinotsuka H., Tanuma S., Powell C.J., Penn D.R. (2015). Calculations of electron inelastic mean free pahs. X. Data for 41 elemental solids over the 50 eV to 200 keV range with the relativistic full Penn algorithm. Surf. Interface Anal..

[59] Lascovich C., Scaglione S. (1994). Comparison among XAES, PEELS and XPS techniques for evaluation of Sp2 percentage in a-C:H. Appl. Surf. Sci..

