# Supplementary material for: Effect of Microwave Treatment in a High Pressure Microwave Reactor on Graphene Oxide Reduction Process—TEM, XRD, Raman, IR and Surface Electron Spectroscopic Studies
Source: Materials (Basel). 2021 Sep 30;14(19):5728. doi: 10.3390/ma14195728 (PMC8510118; doi:10.3390/ma14195728)
Supplement: Supplementary file 1 [file materials-14-05728-s001.zip › materials-1384044-supplementary.pdf]

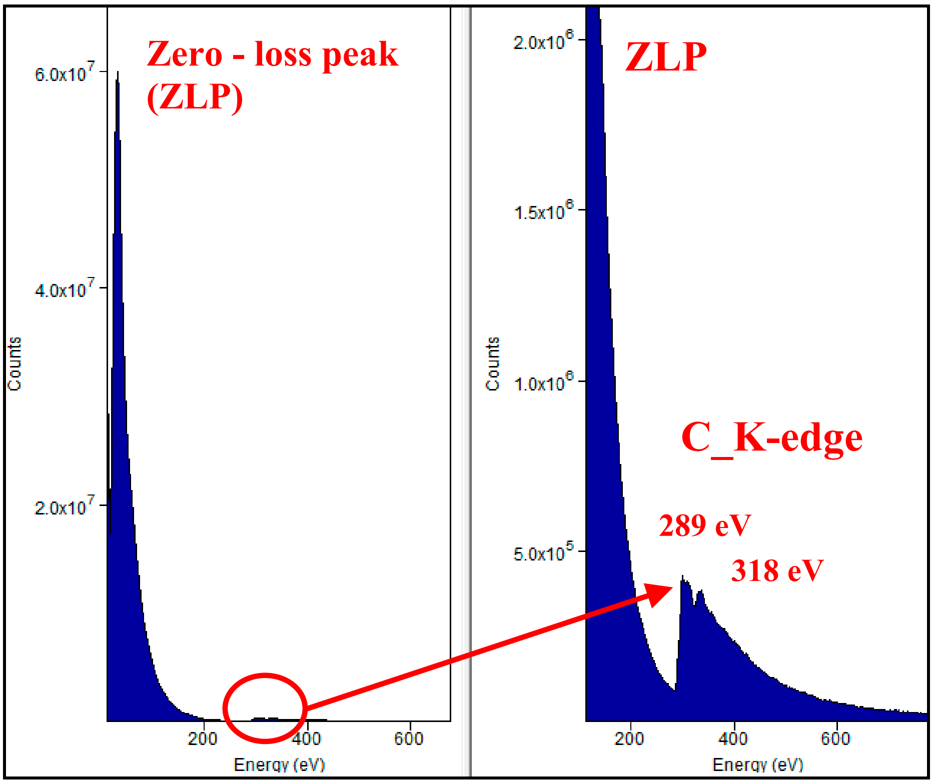

(a)

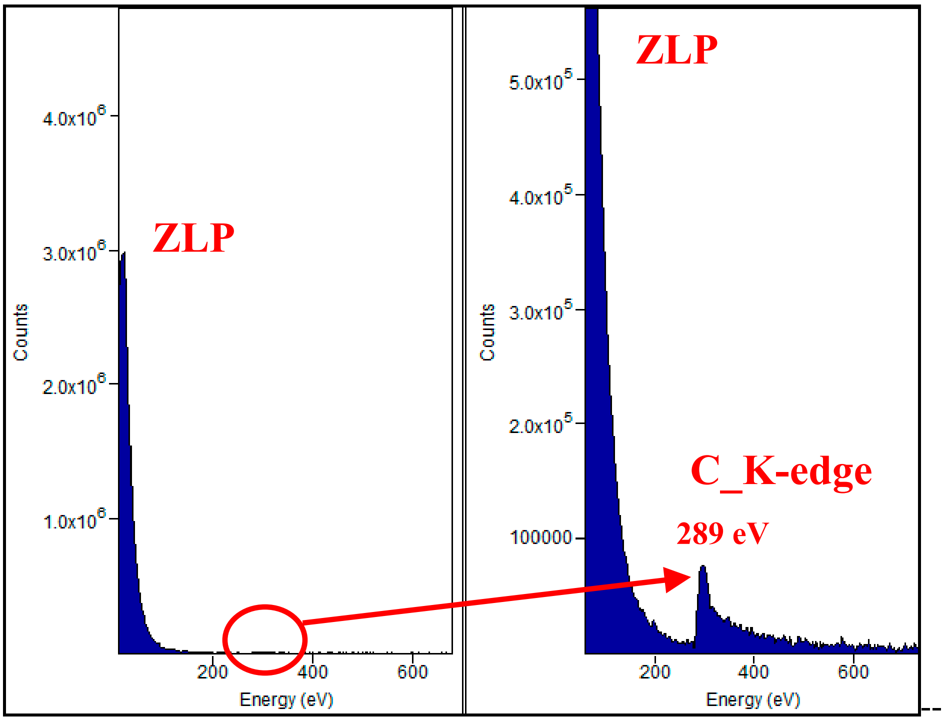

(b)

Figure S1. PEELS spectra of (**a**) rGO-CH_2O [23] and (**b**) rGO-CH_2O-MWT.

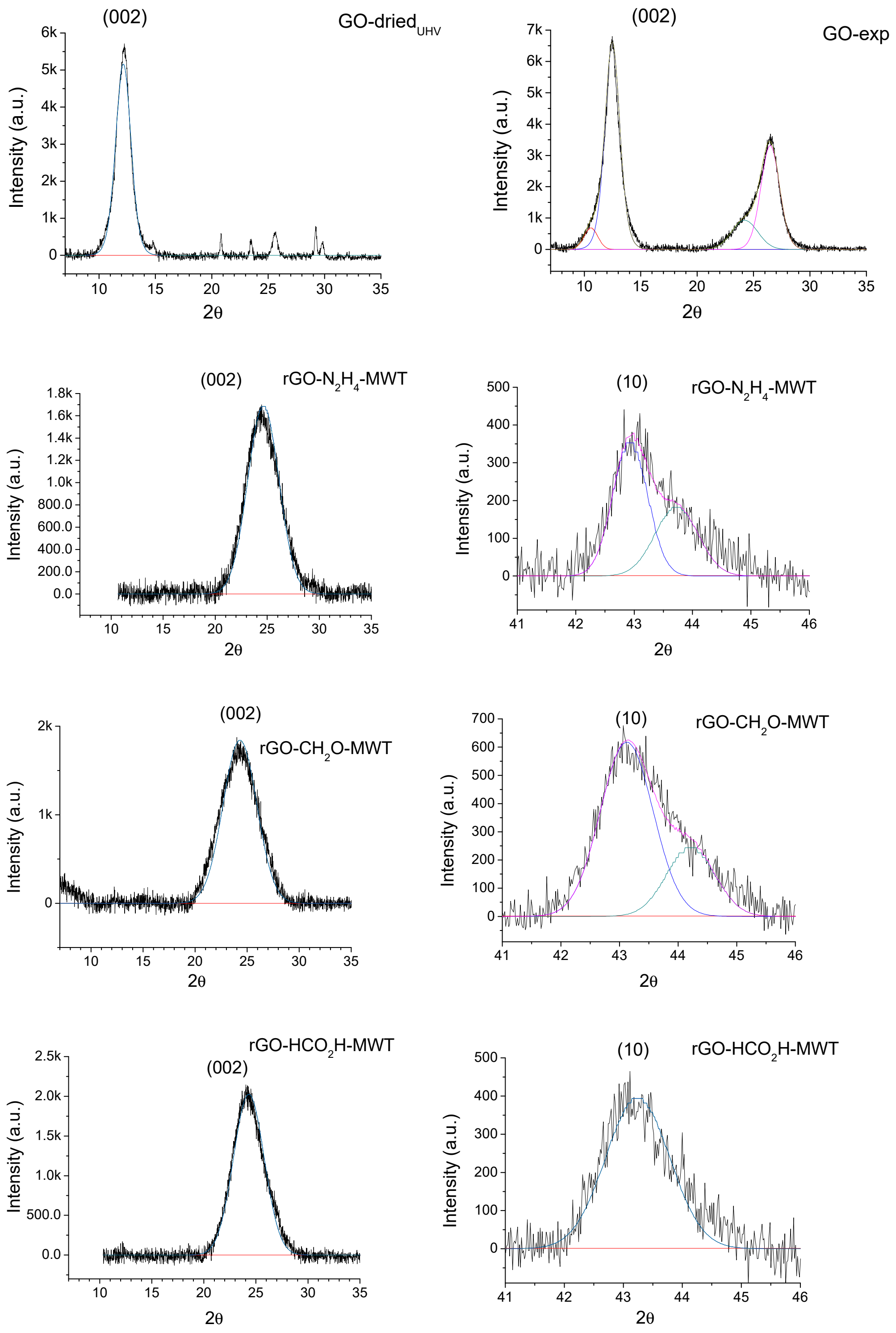

Figure S2. Results of XRD spectra fitting using Pearson7 function for the investigated samples.

Table S1. Parameters of XRD spectra recorded from the investigated samples. These parameters were considered in evaluation of structural properties of GO and rGO like an interlayer distance, *d*, average number of stacking nanostructures, *H*, average number of layers in stacking nanostructurs, *n*, and average diameter of stacking nanostructures, *D*, (Table 1).

Sample	Peak (002)			Peak (10)		
	Area (%)	2θ (deg)	FWHM (deg)	Area (%)	2θ (deg)	FWHM (deg)
*GO-foil	1	12.3384	1.6667			
GO-dried$_{UHV}$	1	12.1533	1.7586			
**rGO-N_2H_4-MWT**	1	24.6274	3.99674	0.66	42.9323	1.5121
				0.34	43.7257	1.5009
GO-exp	0.07549	10.5744	2.49572			
	0.50553	12.4794	1.72772			
	0.10951	24.2124	2.66205			
	0.30948	26.4764	2.11076			
rGO-exp-N_2H_4	0.31	24.2537	0.8838	0.93	42.8600	10.3950
	0.69	26.3226	2.0614	0.07	43.8558	0.8674
*rRGO-CH_2O	1	26.165	2.1610	0.35	42.8002	1.2304
				0.65	43.4971	2.9751
**rGO-CH_2O-MWT**	1	24.3071	3.9355	0.90	43.1234	1.0586
				0.10	44.2152	0.9836
**rGO-HCO_2H-MWT**	1	24.3181	3.71454	1	43.2429	1.8516
*Gr	1	26.6065	0.1990			
*Gr-exp	0.0643	25.4587	1.3452			
	0.7514	26.3283	0.7351			
	0.1844	27.1396	1.3638			

* from [23].

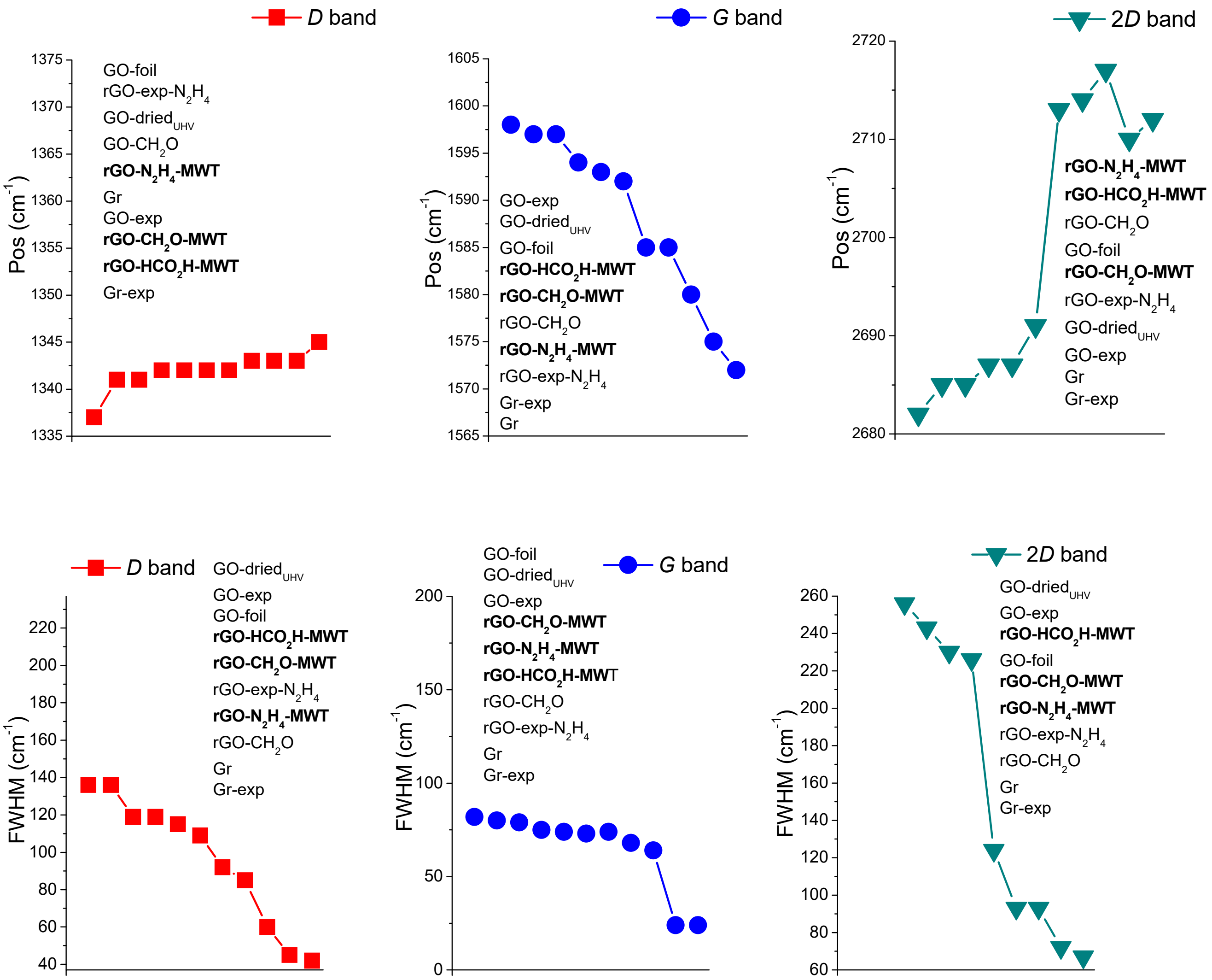

Figure S3. The values of position and FWHM of Raman spectra recorded from the investigated samples indicated from top to bottom in the order starting from GO/rGOs to graphite.

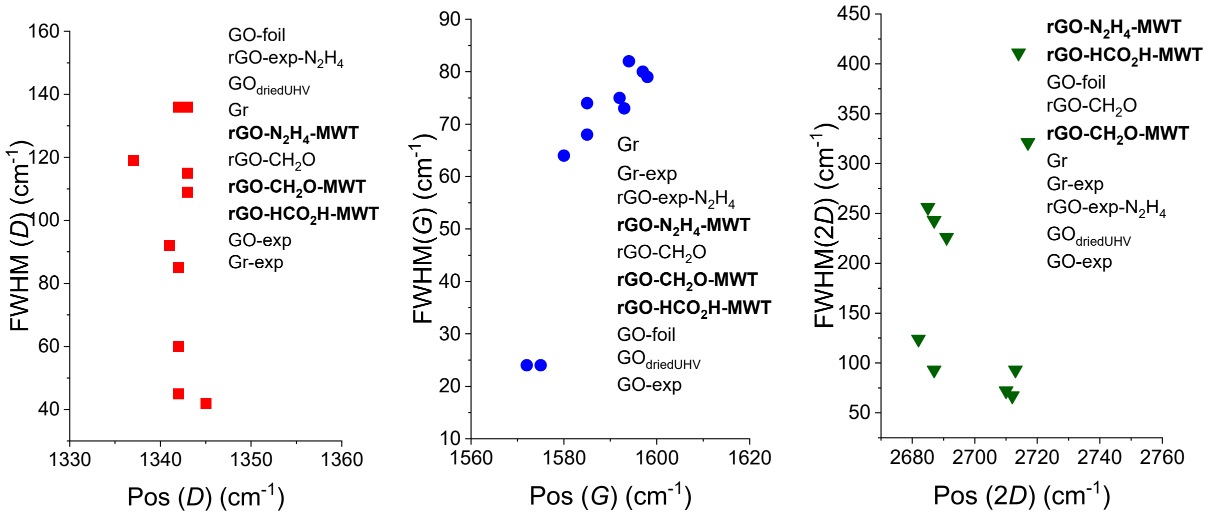

Figure S4. Dependence of Raman *D*, *G* and 2*D* band position on the FWHM in the investigated samples indicated from top to bottom.

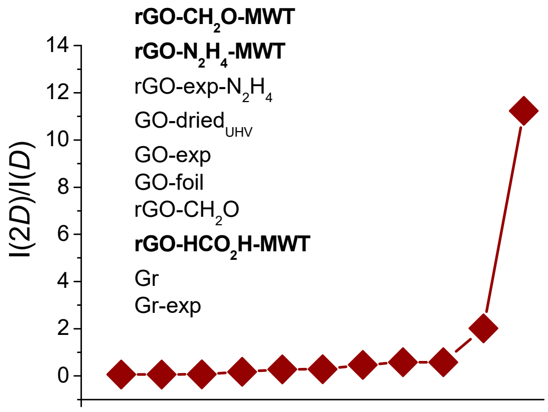

Figure S5. The ratio of intensities of 2*D* to *D* band for the investigated samples indicated from top to bottom in the order of increasing crystallinity.

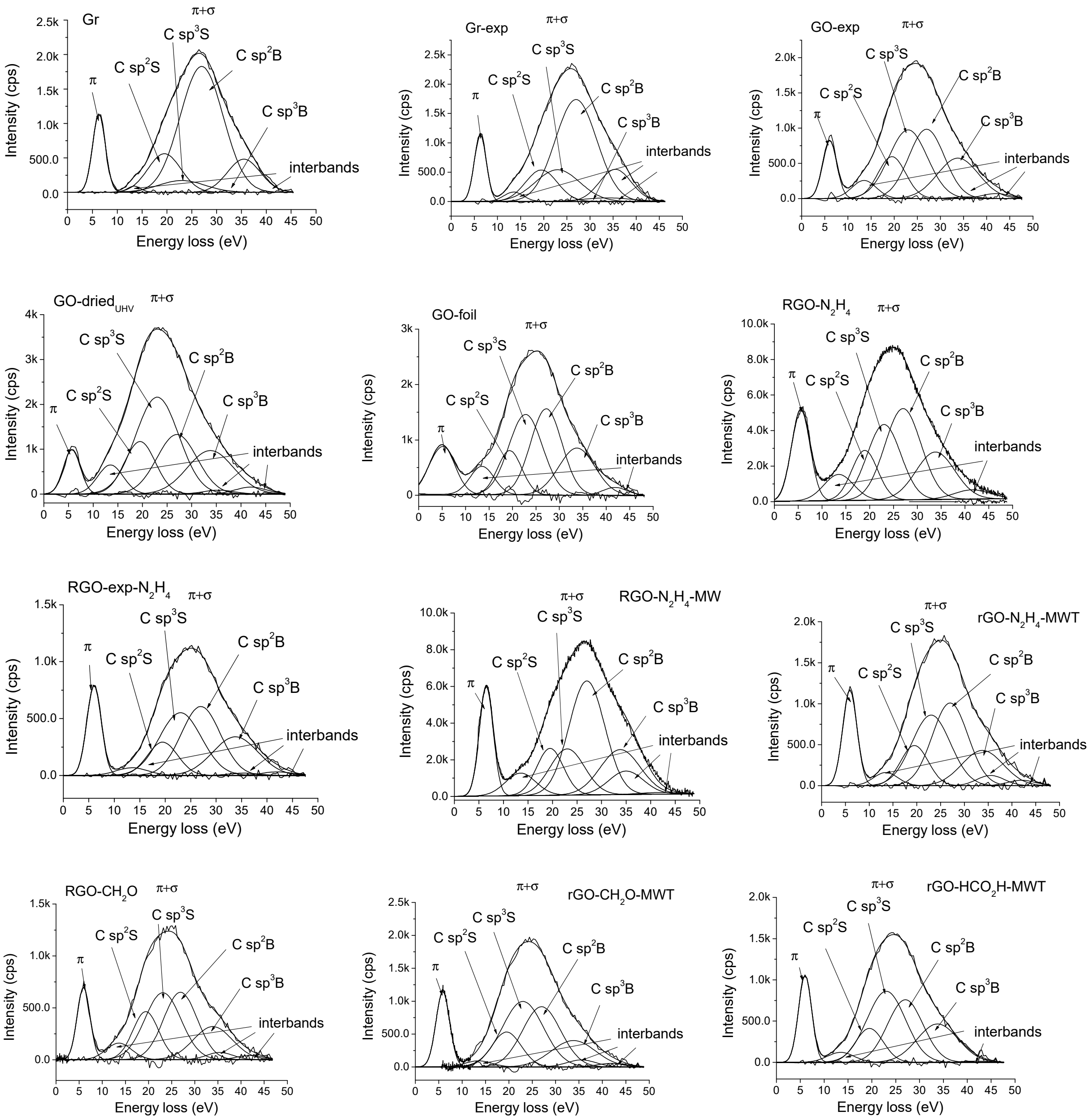

Figure S6. Results of REELS spectra fitting using Gaussian functions. Standard deviation multiplied by 10.

Table S2. Surface atomic concentration of nanocarbon materials determined using XPS.

Sample	Atomic content (at.%)						
	C	O	N	Na	Mn	S	Si
*GO-foil	73.5	25.3	0.7	-	-	0.5	-
GO-dried$_{UHV}$	71.2	27.6	0.3	-	-	-	-
**rGO-N_2H_4	88.2	8.6	-	-	-	3.2	-
**rGO-N_2H_4-MW	85.3	12.1	-	-	-	2.6	-
**rGO-N_2H_4-MWT**	88.0	8.9	3.1		-	-	
GO-exp	69.2	29.9	-	-	0.4	0.5	-
rGO-exp-N_2H_4	86.8	8.8	4.4	-	-	-	-
*rGO-CH_2O	81.3	16.5	-	2.3	-	-	-
***rGO-CH_2O-MWT**	83.2	16.8	-	-	-	-	
**rGO-HCO_2H-MWT**	82.6	17.4	-	-	-	-	
*Gr	96.1	3.9	-	-	-	-	-
*Gr-exp	78.5	16.2	1.3	-	-	3.7	0.3

* from [23], ** [22].

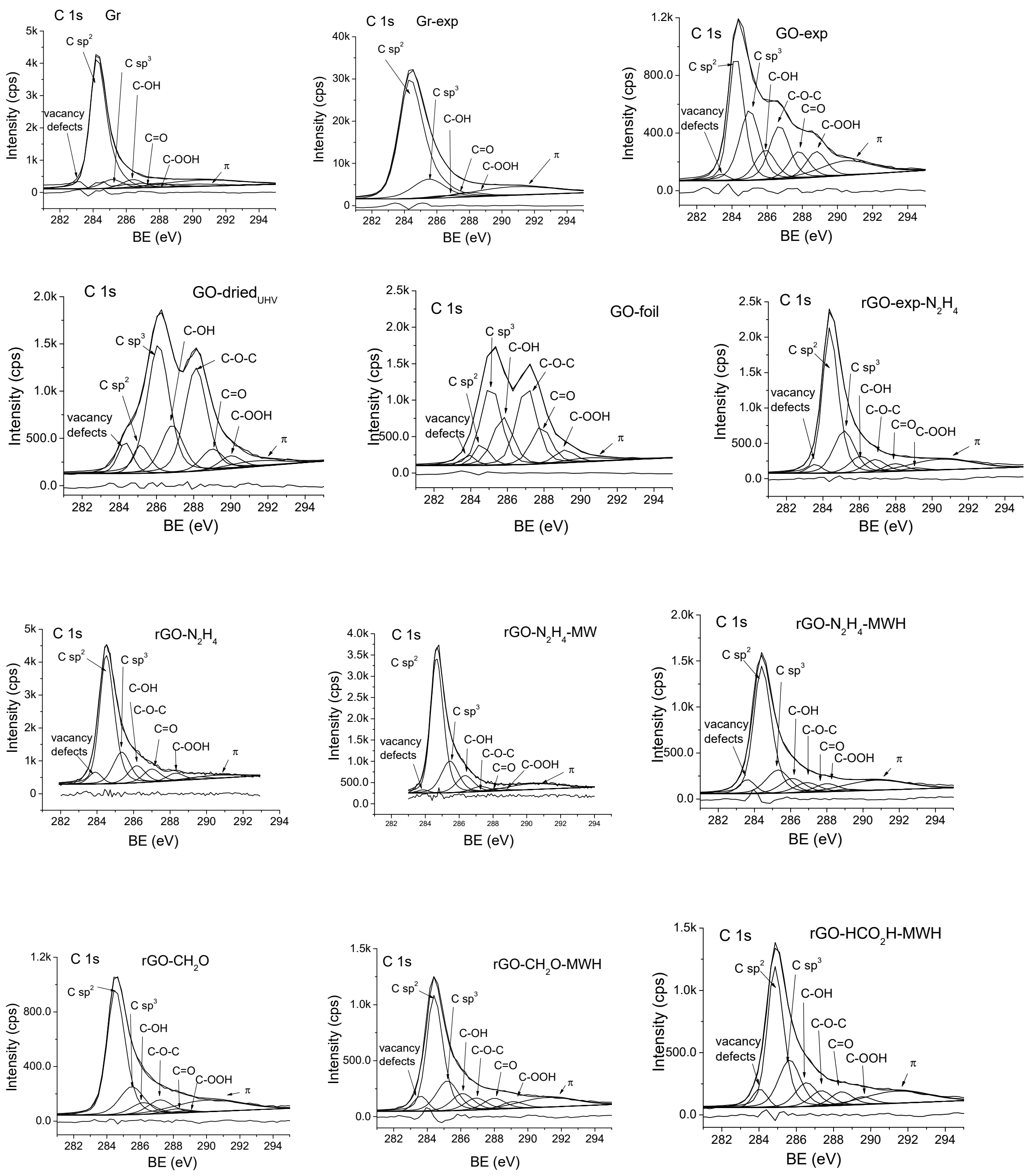

Figure S7. Results of C 1s spectra fitting using asymmetric Gaussian-Lorentzian functions.

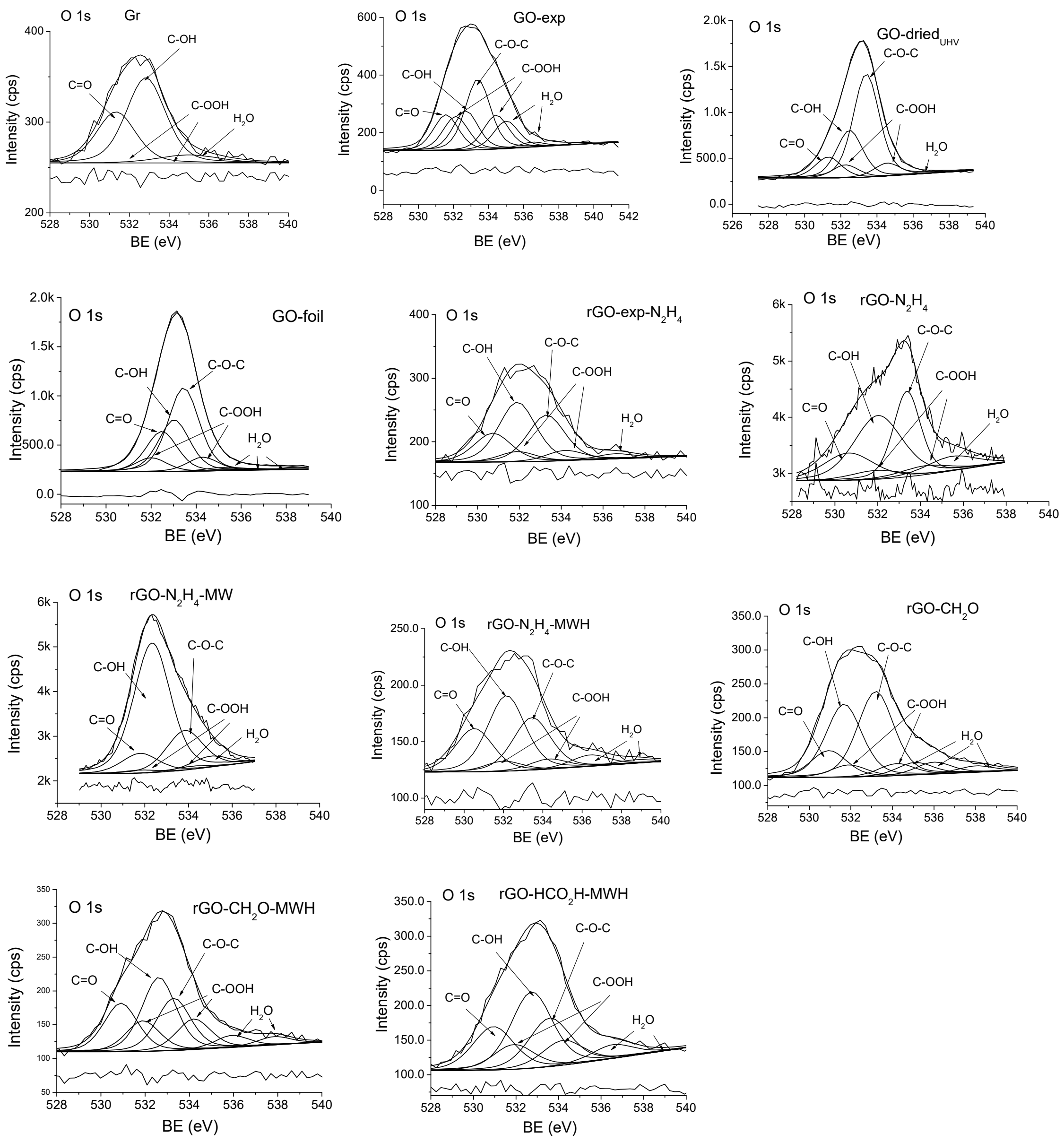

Figure S8. Results of O 1s spectra fitting using Gaussian-Lorentzian functions.

Table S3. Atomic content of C sp2/sp^3 hydridizations and carbon-oxygen groups resulting from C 1s spectra fitting using Gaussian-Lorentzian asymmetric functions.

Sample	C 1s chemical form (at.%)						
	vac. defects 283.7± 0.2 eV	C sp^2 284.5 ±0.1 eV	C sp^3 285.3 ±0.1 eV	C-OH 286.2 ±0.3 eV	C-O-C 287.1 ±0.1 eV	C=O 288.1 ±0.3 eV	C-OOH 289.2 ±0.3 eV
*GO-foil	1.8	4.5	21.0	12.6	20.4	9.8	3.4
GO-dried$_{UHV}$	4.1	4.5	25.6	9.5	20.7	4.2	2.6
**rGO-N_2H_4	4.1	52.4	13.9	7.6	5.7	3.4	1.1
**rGO-N_2H_4-MW	1.1	55.3	16.3	8.5	2.6	4.2	0.2
**rGO-N_2H_4-MWT**	4.9	52.9	12.2	7.5	5.2	4.3	1
GO-exp	1	22.4	15.6	6.6	11.9	6	5.7
rGO-exp-N_2H_4	2.6	49.3	17.9	7	5.4	3.4	1.2
*rGO-CH_2O	0	50.9	14.2	6	7	2.2	1
***rGO-CH_2O-MWT**	4.1	43.1	14	7.9	5.6	5.2	3.3
**rGO-HCO_2H-MWT**	3.9	40	16.9	8.6	5.5	4.9	2.8
*Gr	0	78.4	8.8	4.8	-	4.1	-
*Gr-exp	0	62.6	11.2	1.3	-	2.7	0.7

* from [23]. ** [22].

Table S4. Atomic content of carbon-oxygen groups and water resulting from O 1s spectra fitting using Gaussian-Lorentzian asymmetric functions.

Sample	O 1s chemical form (at.%)					
	C=**O** BE (eV) 531.3±0.3 eV	C-**O**H 532.7 ±0.3 eV	C-**O**-C 533.3 ±0.3 eV	C-**O**OH 531.7 ±0.1 eV	C-O**O**H 534.0 ±0.1 eV	H_2**O** BE (eV) 535.1±0.1 eV, 536.5±0.5 eV, 538.4±0.3 eV
*GO-foil	4.8	6.2	10.0	1.7	1.7	0.4,0.2,0.3
GO-dried$_{UHV}$	2.9	6.6	14.4	1.8	1.8	0.1,0,0
**rGO-N_2H_4	1.4	3.1	2.4	0.5	0.5	0.7,0,0
**rGO-N_2H_4-MW	1.2	7.6	2.3	0.2	0.2	0.6,0,0
**rGO-N_2H_4-MWT**	1.9	3.2	2.3	0.4	0.4	0,0.5,0.2
GO-exp	4.3	4.7	8.6	4.1	4.1	3.4,0.6,0
rGO-exp-N_2H_4	1.6	3.2	2.5	0.6	0.6	0,0.3,0
*rGO-CH_2O	1.9	5	5.9	0.8	0.8	0.9,0.8,0.4
***rGO-CH_2O-MWT**	3.2	4.8	3.4	2	2	0,0.8,0.6
**rGO-HCO_2H-MWT**	3.3	5.7	3.6	1.9	1.9	0,1,0
*Gr	1.7	2.0	-	-	-	0.1,0.1,0

*from [23], **[22].

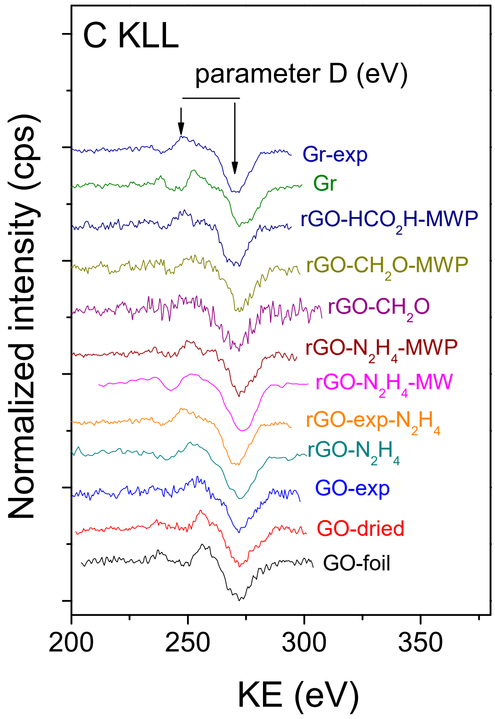

Figure S9. Exemplary first derivative C KLL Auger spectra (smoothing using weighted average with 9 window points) recorded for carbon nanomaterials for evaluation of parameter D.

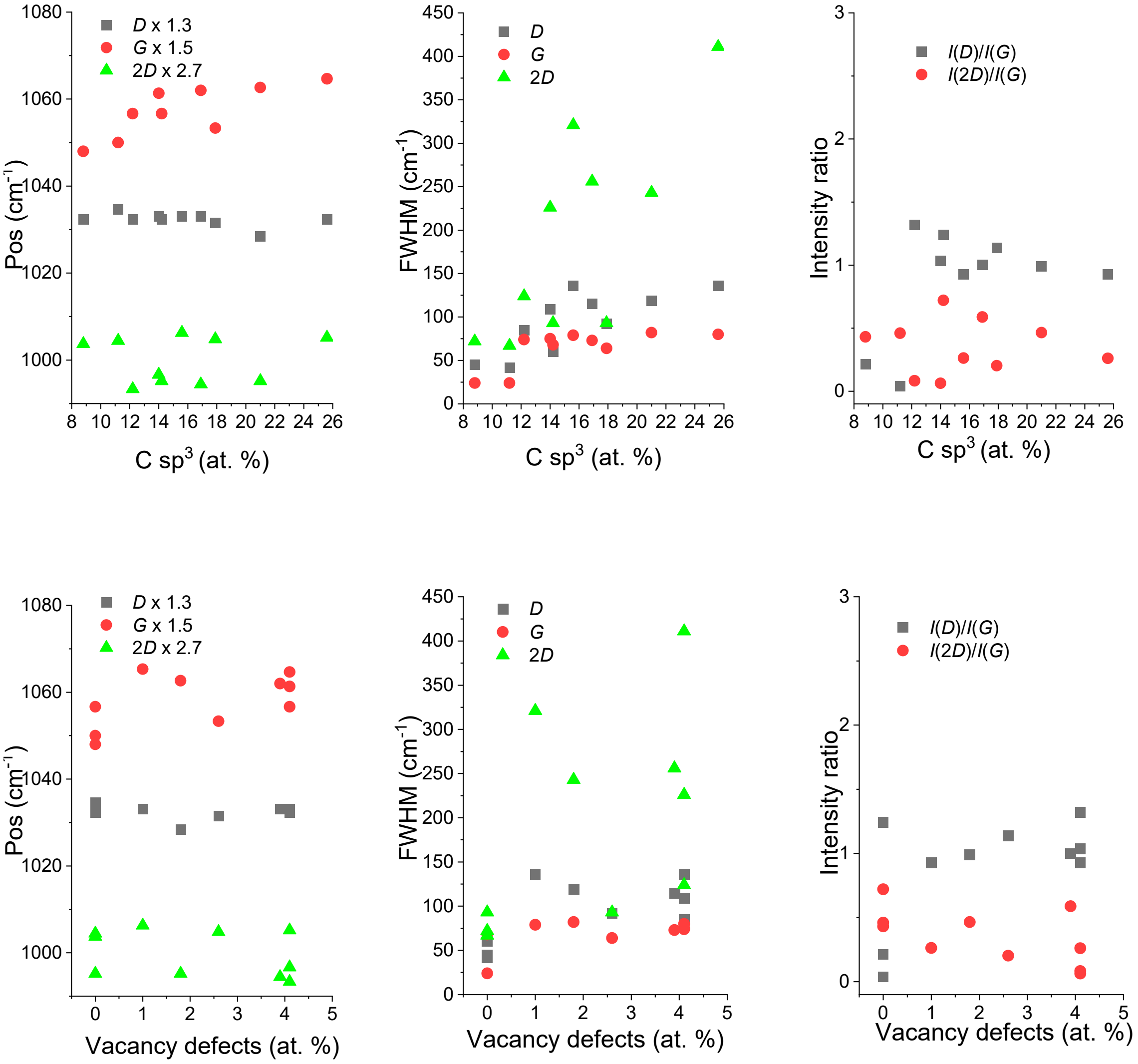

Figure S10. Dependence of C sp³ and vacancy defects on position, FWHM and intensity of Raman spectra for the investigated samples.
